# *Pten* haploinsufficiency causes desynchronized growth of brain areas involved in sensory processing

**DOI:** 10.1016/j.isci.2022.103796

**Published:** 2022-01-19

**Authors:** Amy E. Clipperton-Allen, Hannah Swick, Valentina Botero, Massimiliano Aceti, Jacob Ellegood, Jason P. Lerch, Damon T. Page

**Affiliations:** 1 Department of Neuroscience, The Scripps Research Institute, Jupiter, FL 33458, USA; 2 Doctoral Program in Chemical and Biological Sciences, The Skaggs Graduate School of Chemical and Biological Sciences at Scripps Research, Jupiter, FL 33458, USA; 3 Mouse Imaging Centre, Hospital for Sick Children, Toronto, ON M5T 3H7, Canada; 4 Wellcome Centre for Integrative Neuroimaging, University of Oxford, Oxford, Oxfordshire OX3 9DU, UK

**Keywords:** Behavioral neuroscience, Biological sciences, Imaging anatomy, Neuroscience, Small animal imaging

## Abstract

How changes in brain scaling relate to altered behavior is an important question in neurodevelopmental disorder research. Mice with germline *Pten* haploinsufficiency (*Pten*^+/-^) closely mirror the abnormal brain scaling and behavioral deficits seen in humans with macrocephaly/autism syndrome, which is caused by *PTEN* mutations. We explored whether deviation from normal patterns of growth can predict behavioral abnormalities. Brain regions associated with sensory processing (e.g., pons and inferior colliculus) had the biggest deviations from expected volume. While *Pten*^+/-^ mice showed little or no abnormal behavior on most assays, both sexes showed sensory deficits, including impaired sensorimotor gating and hyporeactivity to high-intensity stimuli. Developmental analysis of this phenotype showed sexual dimorphism for hyporeactivity. Mapping behavioral phenotypes of *Pten*^+/-^ mice onto relevant brain regions suggested abnormal behavior is likely when associated with relatively enlarged brain regions, while unchanged or relatively decreased brain regions have little predictive value.

## Highlights

• Relationship between brain volume and behavioral changes studied in *Pten*^+/-^ mice
• MRI: disproportionately enlarged hindbrain areas involved in acoustic processing
• Acoustic startle testing reveals hyporeactivity to high-intensity stimuli
• Sensory processing deficits precede social deficits during development

## Graphical abstract

**Figure:**
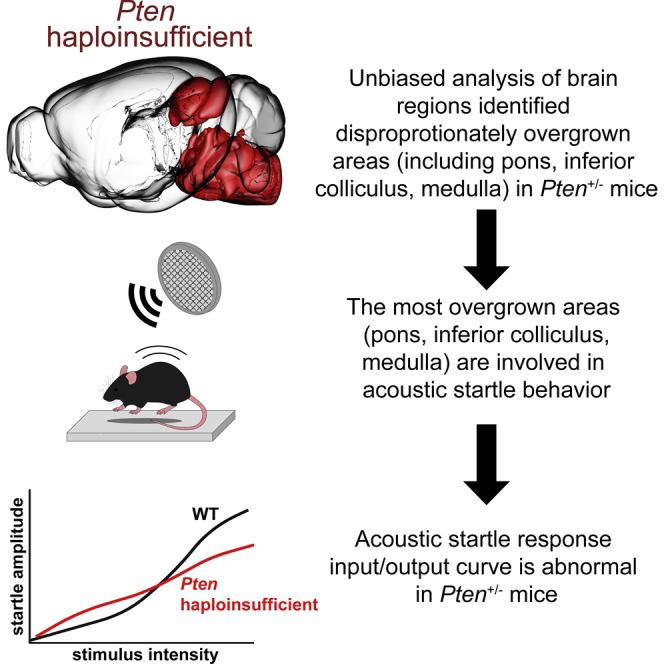

## Introduction

Autism spectrum disorder (ASD), a neurodevelopmental disorder present in 1:39 children in the USA (Baio et al., 2018), is diagnosed based on behavioral symptoms: deficits in social behavior and communication; restricted, repetitive behavior and interests; and sensory abnormalities (APA, 2013). These sensory abnormalities include hypo- or hyper-responsiveness and sensory processing deficits (Marco et al., 2011). As it is a very heterogeneous disorder that presents with a wide range of symptoms and comorbidities, biomarkers that can classify ASD into subgroups are particularly important. One such biomarker is macrocephaly (head circumference >2 SD above normal), which is present in approximately 15%–20% of the population with clinical ASD (Albores-Gallo et al., 2017; Lainhart et al., 2006; Sacco et al., 2015). Mutations in the gene *PTEN* (*Phosphatase and tensin homolog*), which are responsible for macrocephaly/autism syndrome (OMIM #605309), are present in up to 25% of these macrocephalic cases (Butler et al., 2005; Buxbaum et al., 2007; Hobert et al., 2014; Klein et al., 2013; McBride et al., 2010; Varga et al., 2009; Yeung et al., 2017).

In addition to ASD, individuals with *PTEN* mutations also show a number of other behavioral deficits, including impaired motor function and fine motor skills, executive function, processing speed, working memory, and memory recall (Busch et al., 2013, 2019; Frazier et al., 2015). Furthermore, individuals with *PTEN* mutations and ASD show severe sensory functioning impairments, including hyporesponsiveness and acoustic processing deficits (Busch et al., 2019). Anatomically, individuals with *PTEN* mutations show abnormal scaling across brain areas. Although almost all brain areas are increased in volume, when total brain volume is corrected for, white matter and ventricles are relatively enlarged, while gray matter and cerebellar structures are relatively smaller (Frazier et al., 2015). Similar analyses of brain scaling in ASD are routinely performed (e.g., Bigler et al., 2010; Brun et al., 2009; Cleavinger et al., 2008; Herbert et al., 2003; Sears et al., 1999; Sparks et al., 2002), but the behavioral consequences of these alterations in brain scaling are poorly understood.

To address this question, we used a mouse model with germline *Pten* haploinsufficiency, which we have previously shown to be an extremely valid model for the behavioral and neuroanatomical features of human macrocephaly/autism syndrome and *PTEN* mutations (Clipperton-Allen and Page, 2014, 2020; Clipperton-Allen et al., 2019). *Pten* haploinsufficient (*Pten*^+/-^) mice have deficits in social behavior and increased repetitive behavior, but relatively few behavioral phenotypes considering the 20% increase in total brain volume observed in adult mice (Chen et al., 2015; Clipperton-Allen and Page, 2014, 2015, 2020; Page et al., 2009a; Sejourne et al., 2015). Our previous work using MRI revealed that *Pten*^+/-^ mice also show a strikingly similar pattern of brain scaling abnormalities to that of humans with *PTEN* mutations (Clipperton-Allen et al., 2019). We extended the analysis of our MRI dataset to identify regions that show altered growth trajectories, and used this developmental structural analysis as an unbiased screen to suggest additional behaviors that might be altered in *Pten*^+/-^ mice. Those regions showing the biggest deviation from the expected volume, based on the growth trajectory of wild-type littermate (*Pten*^+/+^) controls, include the pons, inferior colliculus, and medulla. Interestingly, these are associated with sensory behavior assays in the acoustic modality, specifically acoustic startle threshold, magnitude, habituation, and pre-pulse inhibition (Carlson and Willott, 1998; Fendt et al., 2001; Koch and Schnitzler, 1997; Koch, 1999; Yeomans and Frankland, 1995). Thus, we utilized the results of behavioral tests, both the novel results included here and those we have previously identified, in *Pten*^+/-^ mice and *Pten*^+/+^ littermate controls to determine whether the pattern of behavioral phenotypes corresponds to, and can be predicted by, the pattern of abnormal brain development.

## Results

### Rate of brain growth in *Pten*^+/-^ mice deviates from controls

**Figure 1:**
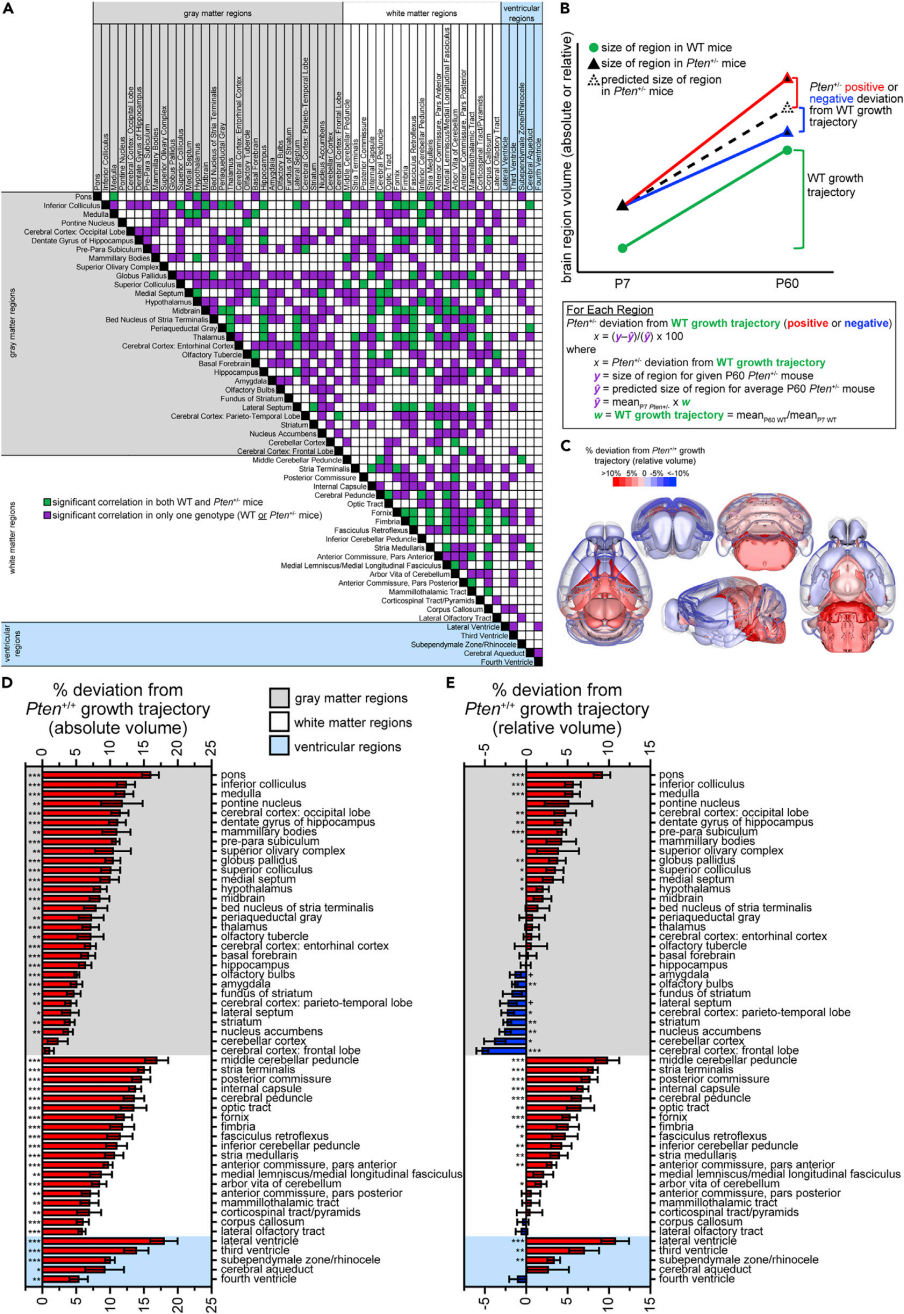
*Pten*^+/-^ mice have abnormal developmental brain region growth trajectories (A) Correlations between brain region growth indices {[(P60 volume for mouse) – (average P7 volume for genotype)]/(average P7 volume for genotype)} within genotypes are present in both genotypes in some regions (green) but not others (purple). (B–E) *Pten*^+/-^ mice have abnormal brain region growth trajectories. We calculated the growth trajectory (B) based on the absolute and relative (percent of total brain) volume for each brain region [(mean_P60 WT region volume_)/(mean_P7 WT region volume_)], and extrapolated the predicted size of each brain region in *Pten*^+/-^ mice based on this trajectory [(mean_P7__*Pten*+/- region volume_) x (WT growth trajectory)]. We then calculated the percent deviation from this predicted size for each mouse {[(region volume) – (predicted region volume)]/(predicted region volume) x 100}. These calculations were performed on absolute (D) and relative (C and E) brain region volumes. (C) Overview of significant *Pten*^+/-^ deviations from predicted relative volume. Red regions are relatively increased, blue regions are relatively decreased, and color intensity indicates the degree of deviation. Cortex is translucent to enable visualization of subcortical structures. (D and E) *Pten*^+/-^ deviation from absolute (D) and relative (E) predicted brain region volume. P7, postnatal day 7; P60, postnatal day 60. The Scalable Brain Atlas (https://scalablebrainatlas.incf.org/composer/?template=ABA_v3; Bakker et al., 2015) was used to make images in (C). Data are represented as mean ± SEM. Black symbols, one-sample *t*-tests vs. expected volume. ∗∗∗ p < 0.001, ∗∗ p < 0.01, ∗ p < 0.05, + p < 0.10. See also Figure S1, Tables S1 and S2.

To gain a high-level overview of altered developmental brain scaling in *Pten*^+/-^ mice, we extended our analysis of our MRI dataset (Clipperton-Allen et al., 2019) to calculate growth indices (normalized increase from postnatal day 7, P7, to P60; see STAR Methods for calculation details). We correlated these indices for all brain regions separately within each genotype, and identified the significant correlations that were consistent or differed between genotypes, as shown in the heatmap in Figure 1A (see Figure S1 for detailed breakdown of correlations in both absolute and relative, i.e., percent of brain, volumes; statistical results are in Table S1). Coordinated growth of areas has been shown (e.g., thalamic projections and visual cortex; see Borello et al., 2018 for a review), and this analysis aimed to identify areas of synchronized growth in *Pten*^+/+^ controls, and determine which of these correlations were maintained in the *Pten*^+/-^ mutant mouse brain. We found that while some correlations were present in both genotypes (green in Figure 1A), the majority were divergent, occurring only in one genotype (purple in Figure 1A; see detailed breakdown in Figure S1, Table S1). This suggests that the pattern of growth differs in many regions, or groups of regions, between *Pten*^+/+^ and *Pten*^+/-^ mice.

To explore this potential desynchrony, and to determine if specific *Pten*^+/-^ brain regions developed at a different rate than in *Pten*^+/+^ littermates, we further examined the deviation of *Pten*^+/-^ from a typical (*Pten*^+/+^) growth trajectory (see Figure 1B). When we looked at this deviation using absolute volume, we found that most brain regions were significantly larger than predicted by the *Pten*^+/+^ growth trajectory; out of 54 brain regions, only the cerebellar cortex and frontal lobe of the cerebral cortex did not show a significantly higher growth trajectory than predicted (see Figure 1D, Table S2). This indicates that brain regions grow more rapidly in *Pten*^+/-^ mice than *Pten*^+/+^ controls, even when differences in P7 brain volume are accounted for. As can be seen in the anatomical overview (Figure 1C), the majority of regions also showed increased growth trajectories for relative volume. However, several regions showed no significant difference from their predicted volumes, and a few gray matter regions, including the frontal lobe, cerebellar cortex, nucleus accumbens, and striatum, were smaller than predicted (see Figure 1E, Table S2).

### Behaviors associated with overgrown brain regions are altered in *Pten*^+/-^ mice

In both absolute and relative (corrected for total brain) volume analyses of deviation from predicted adult brain volume, the most affected (i.e., most overgrown) brain regions were those involved in acoustic startle and pre-pulse inhibition (e.g., pons, inferior colliculus, and medulla; see Figures 1C–1E; Carlson and Willott, 1998; Fendt et al., 2001; Koch and Schnitzler, 1997; Koch, 1999; Yeomans and Frankland, 1995). As we have previously observed deficits in pre-pulse inhibition (Page et al., 2009a), and sensory abnormalities are a key symptom of ASD, we tested *Pten*^+/-^ mice and littermate controls on the acoustic startle threshold (AST) and the acoustic startle habituation/pre-pulse inhibition (ASH/PPI) tests. We also assessed the performance of *Pten*^+/-^ mice on other behaviors associated with regions that were positive, negative, or not deviant from predicted growth trajectories and/or *Pten*^+/+^ volume to explore the relationship between abnormal brain region size and behavioral phenotypes.

#### Acoustic startle threshold test

The acoustic startle response measures sensory reactivity through the whole-body flinch (“startle”) to acoustic stimuli of varying intensities. The neural circuitry involved in this response includes the caudal pontine reticular nucleus (PnC), located within the pons, the inferior colliculus (IC), the medulla, and the superior olivary complex (Carlson and Willott, 1998; Fendt et al., 2001; Koch and Schnitzler, 1997; Koch, 1999; Yeomans and Frankland, 1995). The pons has the largest deviation from relative predicted growth trajectory (see Figure 1E) and the largest difference from *Pten*^+/+^ for relative volume (Clipperton-Allen et al., 2019). In fact, the pons, IC, and medulla are the three gray matter regions with the largest deviation from the *Pten*^+/+^ growth trajectory (see Figure 1E). Other regions involved in this behavior also show large deviations, although they do not reach significance (i.e., superior olivary complex, pontine nucleus; see Figure 1E).

**Figure 2:**
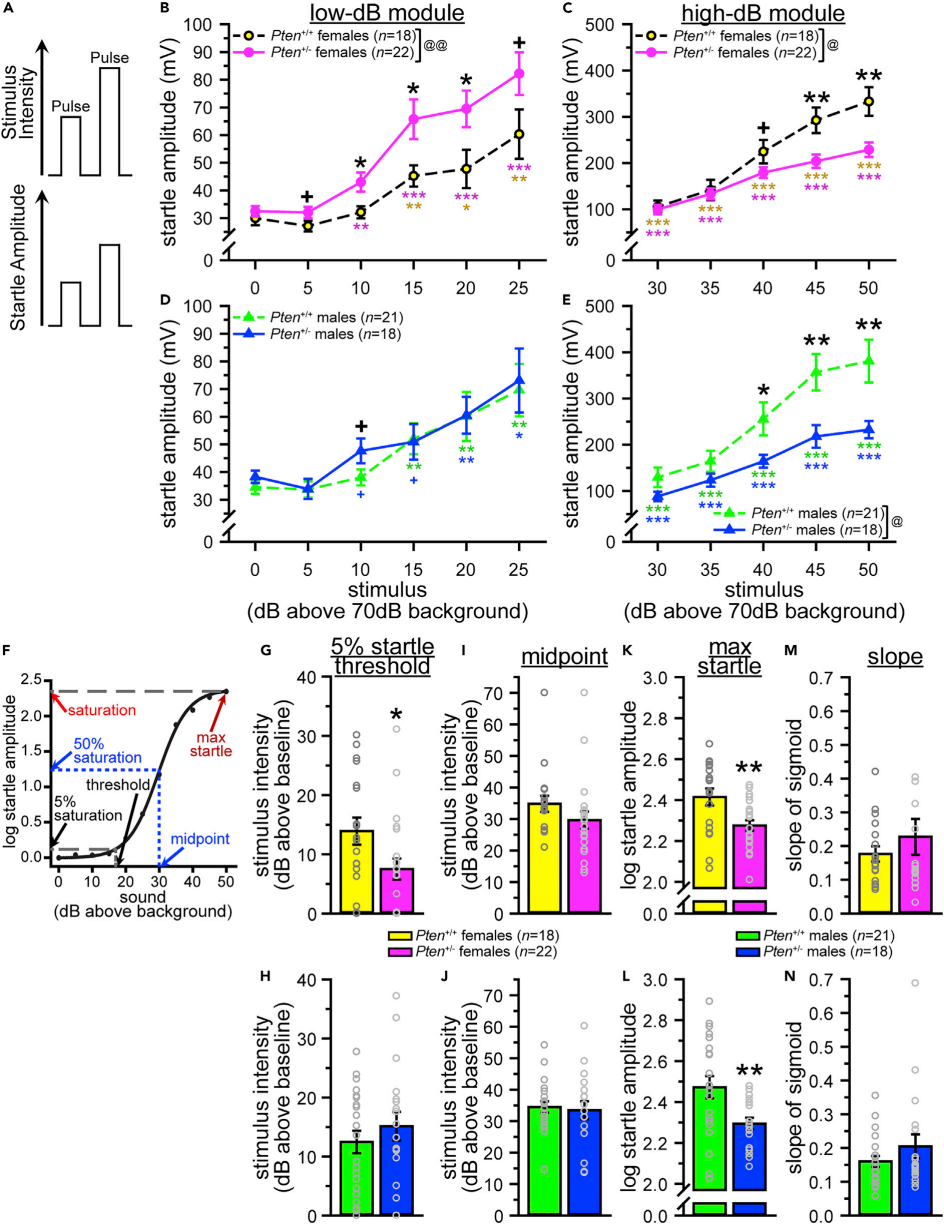
*Pten*^+/-^ mice of both sexes are hyporeactive to acoustic stimuli of high intensity; female *Pten*^+/-^ mice are hypersensitive to acoustic stimuli of low intensity (A) Expected startle responses to white noise stimuli of different intensities above 70 dB background. (B and C) Female *Pten*^+/-^ mice show lower startle thresholds and increased startle amplitude in the low-dB module (B), but lower startle amplitude to stimuli in the high-dB module (C). (D and E) *Pten*^+/-^ males also show decreased startle amplitude to stimuli in the high-dB module (E), with limited evidence of lower startle thresholds and increased startle amplitude in the low-dB module (D). (F–N) Fitting sigmoid curves to each mouse and analyzing the 5% startle threshold (5% of saturation), midpoint (50% of saturation), maximum startle amplitude, and slope of the sigmoid function revealed that *Pten*^+/-^ females (G) but not males (K) show a lower startle threshold, and both sexes of *Pten*^+/-^ mice show lower maximum startle responses (I and M). No genotype differences were found in either sex for midpoint (H and L) or slope (J and N). Data are represented as mean ± SEM. Black symbols, independent-samples *t*-tests between genotypes. Colored symbols, significant startle (paired-samples *t*-tests vs. 0 dB above background). ∗∗∗ p < 0.001, ∗∗ p < 0.01, ∗ p < 0.05, + p < 0.10. Main effect of genotype in two-way mixed-model ANOVAs (genotype x stimulus dB): @@ p < 0.01, @ p < 0.05. See also Figure S2, Table S3.

We used the acoustic startle response measure from the acoustic startle threshold (AST) assay in two ways. First, we analyzed the magnitude of acoustic startle responses (amplitude of startle output in response to white noise inputs of differing intensity), looking at both the input/output (I/O) curve for startle magnitude and the acoustic startle threshold, which we defined as the lowest stimulus intensity to which a mouse will startle significantly more than baseline (see Figure 2A for an example of expected responses to different stimulus intensities). We also analyzed the log-normalized startle response amplitude and fit sigmoid curves to the resulting data as described in Miller et al. (2020). We then used the sigmoid curves to identify the 5% startle threshold (the stimulus intensity that produces 5% of the saturation, which is the maximum startle predicted by the sigmoid function), as well as the midpoint (stimulus intensity that produces 50% of saturation) and slope of the sigmoid (see Figure 2F). As our highest stimulus (50 dB above background) may not have been loud enough to elicit saturation, we also identified the amplitude of the maximum startle response for each mouse.

*Pten*^+/-^ mice of both sexes exhibited lower startle amplitude than *Pten*^+/+^ mice at higher dB (see Figure 2C, females, Figure 2E, males, Table S3). *Pten*^+/-^ females also displayed higher startle amplitudes than *Pten*^+/+^ females at lower stimulus intensities, and a lower startle threshold, significantly startling to a lower dB stimulus than *Pten*^+/+^ females (*Pten*^+/-^, 10 dB above background; *Pten*^+/+^, 15 dB above background; see Figure 2B, Table S3). Male results were less conclusive, as *Pten*^+/-^ male mice showed a trend to lower startle amplitude at one stimulus intensity (10 dB above background, p = 0.071) and a trend to a lower startle threshold than *Pten*^+/+^ males, but the lowest stimulus intensity for a significant startle was higher than that of *Pten*^+/+^ mice (*Pten*^+/+^ threshold, 15 dB above background; *Pten*^+/-^ significant startle at 20 dB above background, trend at 10 dB above background, p = 0.094 and 15 dB above background, p = 0.077; see Figure 2D, Table S3). All mice showed significant startle responses to stimuli 20 dB above background and higher. Similar results were found when we log-normalized the startle amplitude data (Miller et al., 2020), with female *Pten*^+/-^ mice having significantly higher responses at lower stimulus intensities, but lower responses at higher dB above background (Figure S2A; Table S3). Male *Pten*^+/-^ mice showed a main effect of genotype on the log startle amplitude; specifically, the response to high-intensity stimuli was decreased in *Pten*^+/-^ males, as well as to the 5 dB above background stimulus (although only to the level of a trend, p = 0.070, for 35 dB above background; Figure S2B; Table S3).

To further examine the genotype differences in response to the lower- and upper-intensity stimuli, we subdivided the data into low-dB (70–95 dB) and high-dB (100–120 dB) modules and analyzed these using two-way mixed-model ANOVAs (genotype x stimulus dB; see Figure 2, Table S3). Significant main effects of genotype indicated that there were genotype differences across the high-dB module in both sexes (females, Figures 2C and 2G, males, Figures 2E and 2I), but only females showed this difference in the low-dB module (see Figures 2B and 2F, Table S3).

The results of the sigmoid curve analyses (Miller et al., 2020) were consistent with the more traditional method above, showing a significant decrease in the 5% startle threshold in *Pten*^+/-^ females (Figure 2G, Table S3) but not males (Figure 2K, Table S3), and significantly reduced maximum startle amplitude in *Pten*^+/-^ mice of both sexes (females, Figure 2I, males, Figure 2M; Table S3). This was also reflected in the sigmoid curves (see Figures S2C, S2G, and S2I). No genotype differences were found for midpoint (females, Figure 2H, males, Figure 2L; Table S3), slope (females, Figure 2J, males, Figure 2N, Table S3), or model-fitting error (females, Figure S2H, males, Figure S2J, Table S3).

These results indicate that both male and female *Pten*^+/-^ mice show hyporeactivity at higher dB levels relative to *Pten*^+/+^ mice, and female *Pten*^+/-^ mice also show hypersensitivity to lower dB stimuli (startle to lower dB, higher startle amplitude at low dB).

#### Acoustic startle habituation and pre-pulse inhibition test

The pre-pulse inhibition (PPI) assay uses the acoustic startle response measure to examine sensorimotor gating, as well as to assess habituation (ASH) of the mice to repeated presentations of high-intensity (120 dB) stimuli. ASH, like the AST above, is associated with the PnC (pons) and IC, both of which have increased growth trajectories (see Figure 1E) and relative volume (Clipperton-Allen et al., 2019) in *Pten*^+/-^ mice. PPI is also associated with these regions, as well as the superior colliculus (SC; also increased), laterodorsal tegmental nucleus (also in the pons), and midbrain structures including the pedunculopontine tegmental nucleus and the substantia nigra, pars reticulata (Carlson and Willott, 1998; Fendt et al., 2001; Koch and Schnitzler, 1997; Koch, 1999; Yeomans and Frankland, 1995); the midbrain did not show a significant change in relative growth trajectory (see Figure 1E) or relative volume (Clipperton-Allen et al., 2019).

**Figure 3:**
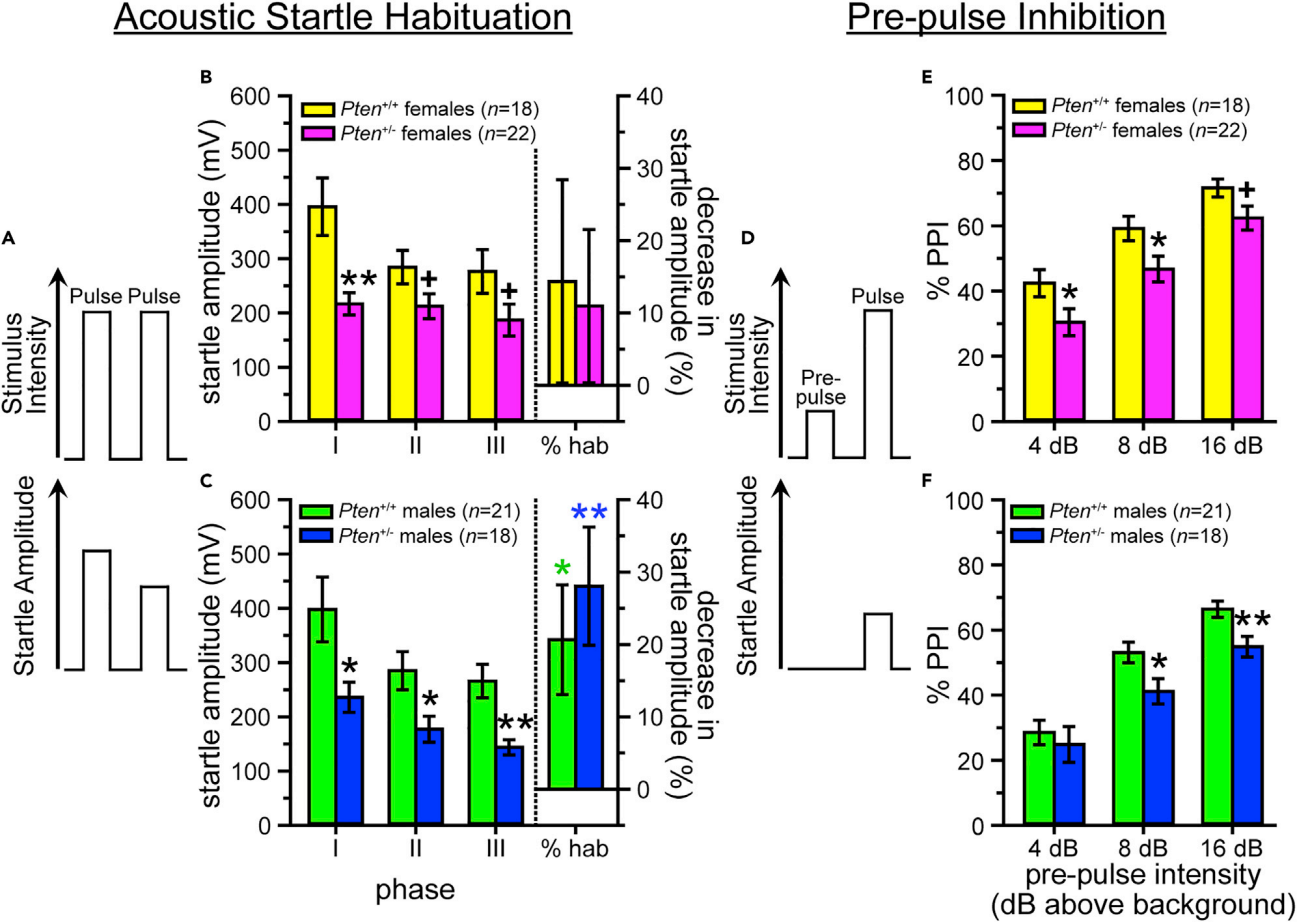
*Pten*^+/-^ mice have lower startle amplitude and decreased pre-pulse inhibition (A and D) Expected startle responses to 120-dB white noise stimuli following repeated presentations (A) and within pre-pulse trials (D). (B and C) Both female (B) and male (C) *Pten*^+/-^ mice startle less than *Pten*^+/+^ mice to a 120-dB white noise pulse during the pre-pulse inhibition (PPI) assay. Neither *Pten*^+/+^ nor *Pten*^+/-^ females show significant habituation [(phase I startle amplitude – phase III startle amplitude)/(phase III startle amplitude) x 100] to the stimulus (B), while both *Pten*^+/+^ and *Pten*^+/-^ males do (C). Phase I, average of 6 stimulus-only presentations prior to PPI phase; phase II, average of 12 stimulus-only presentations during PPI phase; phase III, average of 6 stimulus-only presentations following PPI phase. (E–F) *Pten*^+/-^ mice of both sexes have impaired PPI [(phase II startle amplitude – pre-pulse startle amplitude)/(phase II startle amplitude) x 100], showing decreased inhibition of the startle response following a pre-pulse acoustic stimulus. Data are represented as mean ± SEM. Black symbols, independent-samples *t*-tests between genotypes. Colored symbols, significant habituation (one-sample *t*-tests vs. 0). ∗∗ p < 0.01, ∗ p < 0.05, + p < 0.10. See also Table S3.

Consistent with the high dB hyporeactivity seen in the AST, *Pten*^+/-^ mice of both sexes exhibit lower startle amplitude to the 120-dB stimulus-only trials in all three phases of the ASH/PPI test (phase I: baseline before PPI trials; phase II: interspersed between PPI trials; phase III: after PPI trials; see Figures 3B and 3C, Table S3), although this only reaches the level of a statistical trend in the latter two phases in females (phase II, p = 0.063; phase III, p = 0.074; see Figure 3B, Table S3). Males of both genotypes showed significant habituation over the test (see Figure 3C, Table S3), while females did not (see Figure 3B, Table S3); however, neither sex showed a genotype difference (see Figures 3B and 3C, Table S3). PPI was also reduced in *Pten*^+/-^ mice of both sexes [females: 4 dB, 8 dB, trend (p = 0.059) at 16 dB; males: 8 dB, 16 dB; see Figures 3E and 3F, Table S3].

These data replicate our previous finding of impaired PPI in both sexes (Page et al., 2009a), and, together with the acoustic startle threshold test data above, are the only behavioral phenotypes that have been identified in both sexes in germline *Pten* haploinsufficient mice (see Clipperton-Allen and Page, 2020 for a review). Thus, the finding of similar alterations in both males and females is highly unusual and suggests that this is a particularly penetrant phenotype.

#### Novel object recognition test

**Figure 4:**
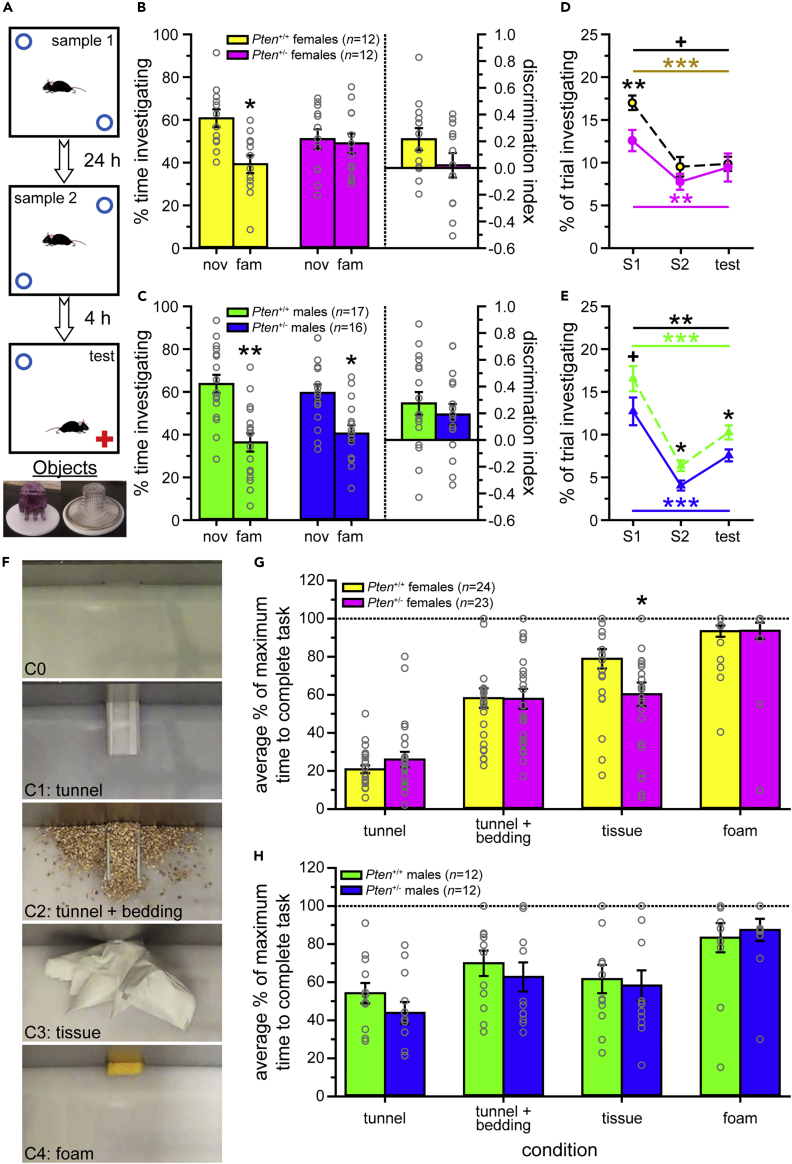
*Pten*^+/-^ females show altered cognitive behavior (A) Schematic of the novel object recognition task. (B and C) Female *Pten*^+/+^ mice (B), and males of both genotypes (C), prefer the novel object during the test phase, while *Pten*^+/-^ females do not (B). Percent time investigating (each object) = {[(time investigating novel or familiar object)]/[(time investigating novel object) + (time investigating familiar object)] x 100}. Discrimination index = {[(time investigating novel object) – (time investigating familiar object)]/[(time investigating novel object) + (time investigating familiar object)] x 100}. (D and E) *Pten*^+/-^ female mice investigate the sample objects less than *Pten*^+/+^ females during the first sample phase (D), while male *Pten*^+/-^ mice spend less of the trial investigating the sample objects across the three phases (E). Nov, novel object; fam, familiar object; S1, sample phase 1; S2, sample phase 2. (F) Examples of the puzzle box conditions. Conditions 1–4 (C1–C4) were each presented three times, while C0 was used for the first and last trials of the experiment. (G and H) Female *Pten*^+/-^ mice performed significantly better on the tissue task (G), but no other genotype differences were observed in either sex (G–H). Data are represented as mean ± SEM. Black symbols, paired-samples *t*-tests between novel and familiar objects within groups (B–C) or independent-samples *t*-tests between genotypes (D–E). Black symbols over lines, main effect of genotype in two-way mixed-model ANOVAs (genotype x phase, D-E). Colored symbols (D–E), change in investigation over phases (one-way within-subjects ANOVAs). Dashed lines (G–H), maximum time to complete task. ∗∗∗ p < 0.001, ∗ p < 0.05, + p < 0.10. See also Figure S3, Tables S4 and S9.

In order to determine the relationship between brain scaling abnormalities and behavior, we tested behaviors associated with brain regions that were relatively increased, relatively decreased, and relatively unchanged. Additionally, little to no testing of non-social recognition memory (particularly without aversive or other external motivation) has been performed on the *Pten*^+/-^ model (Clipperton-Allen and Page, 2020). The novel object recognition assay (NOR) assesses the preference for a novel object, which indicates recognition of the familiar object, as mice will preferentially explore a novel stimulus (e.g., mouse or object) over a familiar one. It also provides a measure of investigation that can show habituation and dishabituation (see Figure 4A). This behavior is associated with the prefrontal and anterior cingulate cortices (PFC and ACC, respectively; part of the frontal lobe in our MRI analysis), the perirhinal cortex (part of the parietal-temporal lobe in our MRI analysis), the entorhinal cortex, and the hippocampus (Cohen and Stackman, 2015; Warburton and Brown, 2015; Weible et al., 2009). *Pten*^+/-^ mice show decreased relative growth trajectory (Figure 1E) and relative volume (Clipperton-Allen et al., 2019) for the frontal and parietal-temporal lobes, but no relative changes in the entorhinal cortex or hippocampus.

Novel Object Preference: Female *Pten*^+/+^ mice showed a significant preference for the novel object, indicating novelty recognition, while female *Pten*^+/-^ mice did not (see Figure 4B, Table S4). In males, both genotypes recognized the familiar object, as indicated by a significant preference to investigate the novel object (see Figure 4C, Table S4).

Total Object Investigation: Investigation times changed with phase in all groups (see Figures 4D–4E, Table S4). In males of both genotypes, this was due to reduced investigation from sample 1 to both sample 2 and test, but increased investigation from sample 2 to test, thus showing a classic habituation/dishabituation pattern (see Figure 4E, Table S4). Both genotypes of female mice also habituated, with reduced investigation from sample 1 to both sample 2 and test, but did not dishabituate (no change in investigation from sample 2 to test; see Figure 4D, Table S4). Interestingly, female *Pten*^+/-^ mice spent less time investigating the objects during sample 1, but not sample 2 or test (trend to main effect of genotype, p = 0.087; significant genotype difference only for sample 1; Figure 4D), while male *Pten*^+/-^ mice spent less time investigating the objects across phases (main effect of genotype; significant genotype differences in sample 2 and test, with a statistical trend during sample 1, p = 0.090; see Figure 4E, Table S4).

These results indicate that female *Pten*^+/-^ mice fail to show a preference for the novel object. While *Pten*^+/-^ females do show reduced investigation, it is only during sample 1, and thus unlikely to explain the recognition impairment. Male *Pten*^+/-^ mice have normal novel object recognition, although they spend less time investigating objects across phases.

#### Puzzle box and open field test

The puzzle box is another cognitive assay, although it is motivated by escaping a brightly lit arena into a dark, enclosed goal box (Ben Abdallah et al., 2011). It assesses cognitive flexibility, as mice need different techniques to solve each of the 4 test conditions (see Figure 4F, Table S9). Unlike NOR, this paradigm is associated with brain regions that have volume (Clipperton-Allen et al., 2019) and growth trajectories (Figure 1E) that are relatively increased (hypothalamus), decreased (ACC and PFC, in the frontal lobe), and unchanged (hippocampus, and thalamus; Ben Abdallah et al., 2011; O'Connor et al., 2014).

Female *Pten*^+/-^ mice solved the tissue obstacle (condition 3) significantly faster than *Pten*^+/+^ females (see Figure 4G, Table S4), and the percent of mice successfully completing the task was significantly higher in *Pten*^+/-^ than *Pten*^+/+^ females for the first two trials of the condition (see Figure S3A, Table S4). No genotype differences were found in any other condition in females (see Figure 4G, S3A, Table S4), and males showed no difference in completion time or percent completing in any condition or trial (see Figure 4H, S3B, Table S4). Additionally, no group showed a change between baseline and final “no obstacle” (condition 0) trials, suggesting that there was no decrease in motivation or habituation to the arena and/or procedure across testing (see Figures S3C–S3D, Table S4).

To ensure that the improved performance by *Pten*^+/-^ females on the tissue condition, and/or the lack of behavioral phenotypes, were not due to confounding locomotor or anxiety phenotypes, the open field test (OFT) was administered to these mice. There were no genotype differences in percent of time spent in the center (females, Figure S3E, males, Figure S3F), distance traveled (females, Figure S3I, males, Figure S3J), or velocity (females, Figure S3K, males, Figure S3L) in either sex, and all groups spent significantly more time in thigmotaxis than in the center (females, Figure S3G, males, Figure S3H, Table S4). Thus, there was no evidence of anxiety or locomotor phenotypes that could confound the results.

Overall, male *Pten*^+/-^ mice did not differ from *Pten*^+/+^ males on the puzzle box or OFT. *Pten*^+/-^ females showed improved performance on a single condition, which could not be explained by differences in locomotion or anxiety.

#### Morris water maze

Memory impairments have been found in some individuals with ASD and *PTEN* mutations (Busch et al., 2013, 2019; Frazier et al., 2015), so we tested *Pten*^+/-^ mice and *Pten*^+/+^ littermates on the Morris water maze (MWM). This task assesses spatial learning and memory; by including reversal learning, we also measure perseveration, an aspect of the restricted, repetitive interests criterion for ASD (APA, 2013). The MWM is associated with several of the same regions as the NOR above, including the PFC and perirhinal cortex in the relatively decreased frontal and parieto-temporal lobes, respectively, and the unchanged entorhinal cortex and hippocampus, as well as the striatum and cerebellum (D'Hooge and De Deyn, 2001; Miyoshi et al., 2012; Morris et al., 1982; Pooters et al., 2015; Steffenach et al., 2005), which show decreased growth trajectories (Figure 1E) but not relative volume (Clipperton-Allen et al., 2019).

**Figure 5:**
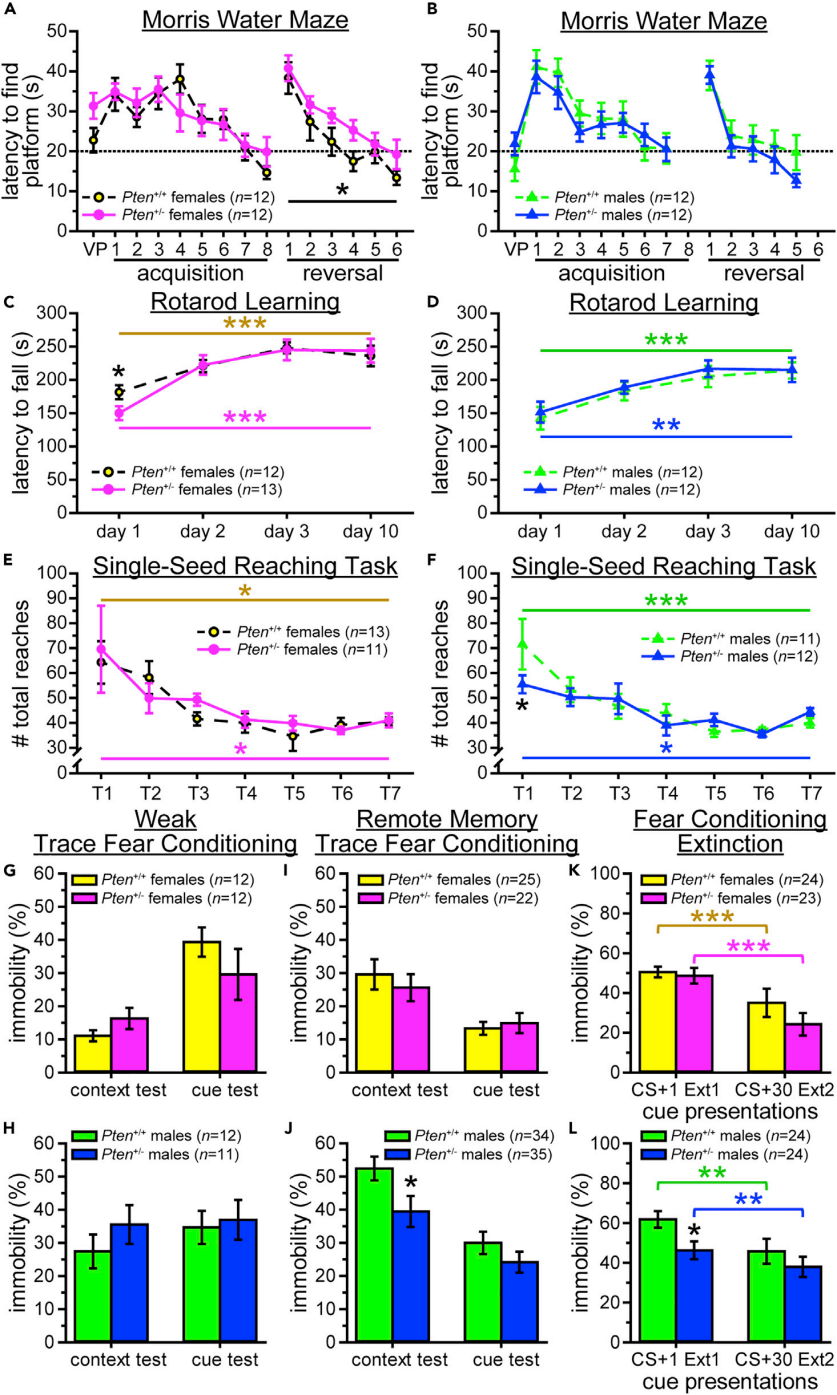
*Pten*^+/-^ mice were normal on the majority of behavioral assays (A and B) Latency to find platform in Morris water maze improved over acquisition and reversal training in *Pten*^+/+^ and *Pten*^+/-^ female (A) and male (B) mice. VP, visual platform test. Dashed line, acquisition or reversal criterion. (C and D) All female (C) and male (D) mice improved their latency to fall across testing days in the rotarod learning assay. (E and F) Female (E) and male (F) mice of both genotypes showed a reduction in the number of reaches to criterion across training in the single-seed reaching task. T1-T7, training days 1–7. (G–L) Normal fear conditioning, including weak trace fear conditioning (G–H), remote memory for trace fear conditioning (I–J), and cued fear conditioning extinction (K–L), was observed in female (G,I, and K) and male (H,J, and L) *Pten*^+/-^ mice, except that *Pten*^+/-^ males showed impaired remote contextual memory (J). Ext bin, average of 6 extinction trials. Data are represented as mean ± SEM. Black symbols, main effect of genotype in two-way mixed-model ANOVAs (genotype x training day, A–B), independent-samples *t*-tests between genotypes (C–D, G–L), or Sidak *post hoc* tests between genotypes from two-way mixed-model ANOVAs (genotype x training day, E-F). Colored symbols, change over time (one-way within-subjects ANOVAs, C–F, or paired-samples *t*-tests, G–L). ∗∗∗ p < 0.001, ∗ p < 0.05, + p < 0.10. See also Figures S4–S6, Tables S4, S5 and S6.

The performance of *Pten*^+/-^ mice on this task was mostly normal. All groups improved over training (significant main effects of day), during both acquisition and reversal learning, showing reduced latency to find the platform (see Figures 5A and 5B, Table S4) and distance traveled (see Figures S4A and S4B, Table S4), and increases in the percent of trials that were successful (see Figures S4C and S4D, Table S4). All groups also showed improvement on probe trial measures, including all measures across acquisition training and many during reversal training (significant main effects of day; see Figures S4E–S4P, Table S4). However, there were a few genotype differences on the probe trial measures. During reversal training probe trials, female *Pten*^+/-^ mice spent less time in the platform quadrant (see Figure S4K, Table S4), with a trend to being farther from the platform (p = 0.083; see Figure S4E, Table S4). Additionally, when compared to chance, female *Pten*^+/+^ mice spent more time in the platform quadrant during all but the first probe trial in both training phases, while *Pten*^+/-^ females only showed trends to spending more than chance amounts of time in the platform quadrant during the final probe trial of both acquisition and reversal training (all p < 0.091; see Figure S4K, Table S4). Interestingly, the males showed a different pattern of results: *Pten*^+/-^ males spent more than chance amounts of time in the platform quadrant during all but the first acquisition probe trial, while *Pten*^+/+^ male controls were only in the platform quadrant more than chance during the third and fourth acquisition probe trials (see Figure S4L, Table S4).

These results suggest that female *Pten*^+/-^ mice may have subtle spatial memory deficits, while male *Pten*^+/-^ mice may perform slightly better than *Pten*^+/+^, particularly for spatial memory during reversal training.

#### Rotarod learning

We have previously shown that *Pten*^+/-^ mice perform normally on the rotarod test, but since individuals with *PTEN* mutations and ASD have been shown to have motor impairments (Busch et al., 2013, 2019; Frazier et al., 2015), and the rotarod task is associated with brain regions that have relatively decreased (motor and prefrontal cortices, amygdala, striatum, and cerebellum; Cao et al., 2015; Kupferschmidt et al., 2017; Scholz et al., 2015) or unchanged (hippocampus and thalamus; Sakayori et al., 2019; Scholz et al., 2015) growth trajectories (Figure 1E) and adult brain volume (Clipperton-Allen et al., 2019), we wanted to determine whether *Pten*^+/-^ mice showed normal motor learning, specifically in the rate of improvement over several days.

All groups improved over time, with the only genotype difference being that *Pten*^+/-^ females had a shorter latency to fall on the first day of training (see Figures 5C and 5D, Table S5). Thus, *Pten*^+/-^ mice show normal learning and memory for this motor behavior.

#### Single-seed reaching task

Fine motor impairments have been observed in humans with *PTEN* mutations and ASD (Busch et al., 2013, 2019; Frazier et al., 2015). To examine the effect of *Pten*^+/-^ mutation on fine motor skills, mice were tested on the single-seed reaching task (SSRT). Like the rotarod learning assay above, the SSRT is associated with regions showing decreased relative growth trajectories (Figure 1E) and volumes (Clipperton-Allen et al., 2019; i.e., motor cortex, striatum, and cerebellum), as well as the thalamus, which is not significantly changed in relative volume (Clipperton-Allen et al., 2019) or growth trajectory (Figure 1E), and the medulla, which is relatively increased on both measures (Figure 1E; Clipperton-Allen et al., 2019; Azim and Alstermark, 2015; Chen et al., 2014; Esposito et al., 2014; Lopez-Huerta et al., 2016; Ruder et al., 2021; Sakayori et al., 2019; Xu et al., 2009).

All mice improved over training, as shown by the decreased number of reaches (see Figures 5E–5F, Table S5). Female *Pten*^+/+^ mice also improved their success rates over time (see Figures S5A and S5C, Table S5), with significant increases in the percent successful attempts and successful attempts/min, while *Pten*^+/-^ females only showed a trend to increased successes/min (p = 0.063). Both genotypes of male mice improved their success/min rate, but only *Pten*^+/-^ males showed a trend to improving the percent successful attempts (p = 0.084; see Figures S5B and S5D, Table S5). Male *Pten*^+/-^ mice made fewer total reaches on training day 1, but no subsequent days (see Figure 5F, Table S5).

To get a better sense of the pattern of response types, we calculated the average number of successes, drops, hits, and misses, as well as “uncounted” reaches, for each sex and genotype. Planned comparisons found a few genotype differences in selected behaviors on specific training days, but no clear deficits or improvements emerged (see Figures S5E and S5F, Table S5). Thus, performance on this fine motor skill assay was largely normal in *Pten*^+/-^ mice.

As mice were food restricted during the SSRT assay in order to increase the salience of the millet seed rewards, their weights were monitored throughout the experiment. Females showed no genotype differences in baseline free-feeding weight or the percent of free-feeding weight at any point during the experiment, and no change in the percent of free-feeding weight across training (see Figure S5G, Table S5. However, males of both genotypes increased their percent free-feeding weight across the experiment (see Figure S5H, Table S5). Interestingly, *Pten*^+/-^ males maintained a higher percent of free-feeding weight than *Pten*^+/+^ males across the experiment, despite showing no difference in initial free-feeding weight (see Figure S5H, Table S5) and copious previous evidence of no difference in body mass between *Pten*^+/-^ and *Pten*^+/-^ mice (e.g., Chen et al., 2015; Clipperton-Allen and Page, 2014; Clipperton-Allen et al., 2019; Page et al., 2009a).

Data from this experiment indicate that, like gross motor learning on the rotarod, *Pten*^+/-^ mice did not perform abnormally on this fine motor skill assay.

#### Fear conditioning

Previously, we have shown minimal genotype differences on trace fear conditioning. We wondered if making the task more challenging, by using weak trace fear conditioning (WFC) that involved only a single pairing between the conditioned stimulus (CS; context or cue tone) and unconditioned stimulus (US; mild footshock), or by testing long-term memory (remote memory for trace fear conditioning, RMFC) using a 30 day delay between conditioning and test, would reveal more subtle phenotypes. We also wanted to use cued fear conditioning extinction (FCExt) to assess perseveration and extinction in the *Pten*^+/-^ mutant mice. All three fear conditioning paradigms have some brain regions in common, specifically the ACC in the frontal lobe, which has decreased relative growth trajectory (Figure 1E) and relative volume (Clipperton-Allen et al., 2019) in *Pten*^+/-^ mice, the amygdala (which has a trend to decreased growth trajectory, p = 0.082; Figure 1E) and the thalamus (which is unchanged; Figure 1E; Clipperton-Allen et al., 2019; Han et al., 2003; LeDoux, 2000; Mamiya et al., 2009; Vetere et al., 2011; Vetere et al., 2012; Wheeler et al., 2013). While the trace fear conditioning paradigms (WFC and RMFC) have other unchanged regions in common (hippocampus and entorhinal cortex; Figure 1E; Clipperton-Allen et al., 2019), and both RMFC and FCExt involve the PFC in the frontal lobe, the only region with a relatively increased growth trajectory (Figure 1E) implicated in any of the fear conditioning paradigms is the hypothalamus, which is specific to the RMFC (Hwang et al., 2010; Kitamura, 2017; LeDoux, 2000; Lugo et al., 2014; Mamiya et al., 2009; Pattwell et al., 2012; Runyan et al., 2004; Vetere et al., 2011, 2012; Wheeler et al., 2013).

Weak Trace Fear Conditioning: No genotype differences were found in either sex, and all groups were successfully conditioned, as shown by significant differences between baseline and test freezing for both context and cue conditioning (see Figures 5G, 5H, S6A, and S6B, Table S6).

**Figure 6:**
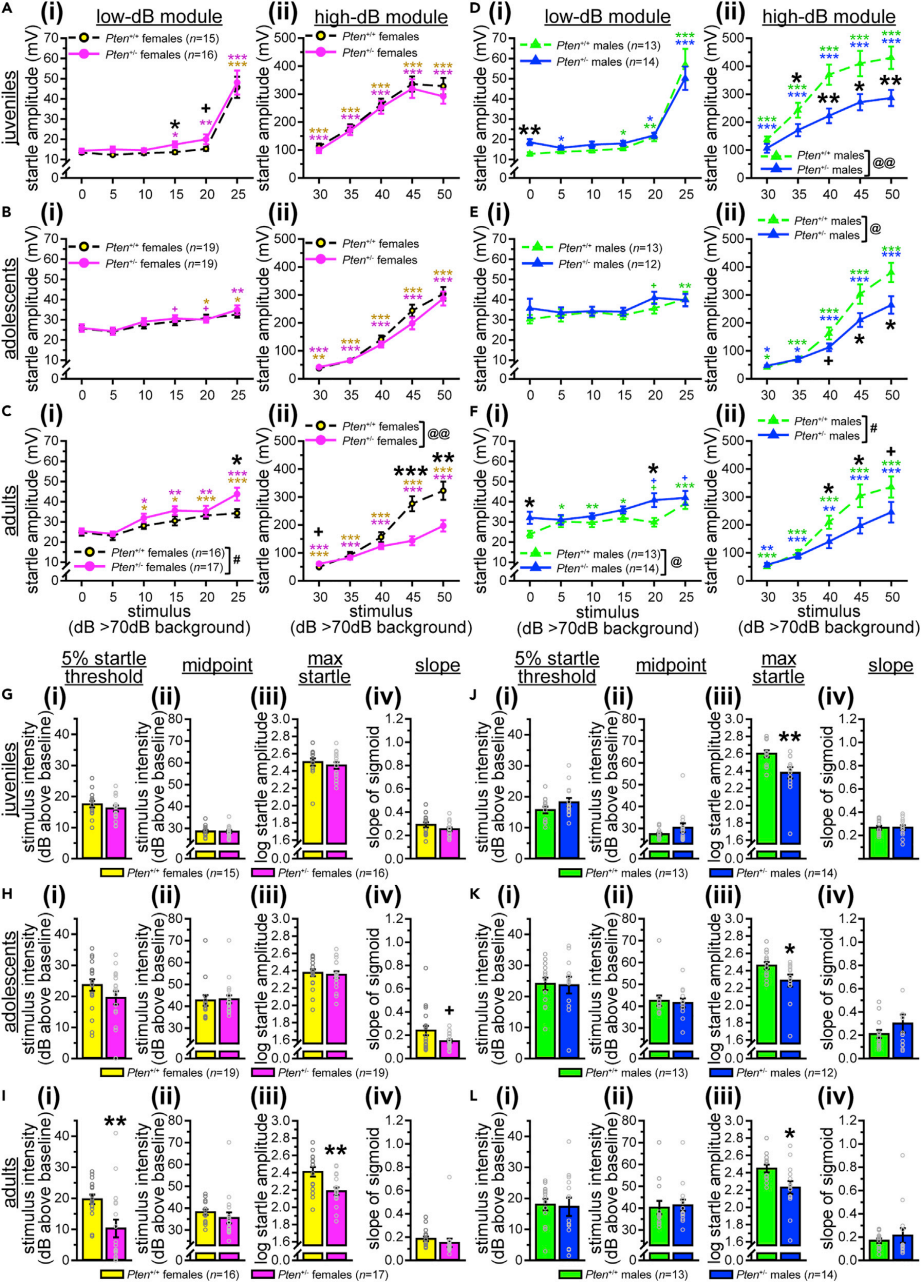
Male and female juvenile *Pten*^+/-^ mice show different aspects of adult acoustic stimuli threshold phenotypes (A–C) Female *Pten*^+/-^ mice show hypersensitivity in the low-dB module as juveniles [A(i)) and adults (C(i)], with limited differences as adolescents [postnatal day 45, B(i)]. In the high-dB module, *Pten*^+/-^ females only show hyporeactivity in adulthood [C(ii)], not as juveniles [A(ii)] or adolescents [B(ii)]. (D–F) *Pten*^+/-^ males express hyporeactivity in the high-dB module throughout development [juveniles, D(ii); adolescents, E(ii); adults, F(ii)], but only show limited evidence of hypersensitivity in the low-dB module as adults [F(i)], with no genotype differences as juveniles [D(i)] or adolescents [E(i)]. (G–L) Fitting sigmoid curves to each mouse and analyzing the 5% startle threshold (5% of saturation), midpoint (50% of saturation), maximum startle amplitude, and slope of the sigmoid revealed that only adult *Pten*^+/-^ females showed significantly lower 5% startle threshold [I(i)] and maximum startle amplitude [I(iii)]. No other significant genotype differences were observed in juvenile (G), adolescent (H), or adult (I) female mice, although there was a trend to a lower sigmoid slope in the adolescent *Pten*^+/-^ females [H(iv)]. Male *Pten*^+/-^ mice showed decreased maximum startle amplitude at all ages [juvenile, J(iii); adolescent, K(iii); adult, L(iii)], but no other genotype differences. Data are represented as mean ± SEM. Black symbols, independent-samples *t*-tests between genotypes. Colored symbols, significant startle (paired-samples *t*-tests vs. 0 dB above background). ∗∗∗ p < 0.001, ∗∗ p < 0.01, ∗ p < 0.05, + p < 0.10. Main effect of genotype in two-way mixed-model ANOVAs (genotype x stimulus dB): @@ p < 0.01, @ p < 0.05, # p < 0.10. See also Figure S8, Table S7.

Remote Memory for Trace Fear Conditioning: As in WFC, females had no genotype differences, with both groups showing evidence of conditioning (significant baseline-test differences for both context and cue conditioning; see Figure 5I, S6C, Table S6). Males, however, did show a genotype difference in the context test, with *Pten*^+/-^ males freezing less than *Pten*^+/+^ males (see Figure 6, Figure 5J and 6D; Table S6). Despite this, males of both genotypes did successfully condition (significant baseline-test differences for context and cue conditioning; see Figure S6D, Table S6).

Cued Fear Conditioning Extinction: All groups extinguished, as shown by decreased freezing across the 10 bins of extinction trials (see Figures S6G and S6H), and from the first to the last cue presentation (see Figures 6K and 6L, Table S6). In females, no genotype differences were found on the first or last cue presentation or bin (Figures 5K, S6E, Table S6), extinction score (Figure S6G, Table S6), or recall test (Figure S6I, Table S6). Males also showed no genotype differences for first cue bin (Figure 6F, Table S6), last cue presentation and bin (Figure 5L, S6F, Table S6), extinction score (Figure S6H, Table S6), and recall test (Figure S6J, Table S6). However, *Pten*^+/-^ males did not show a significant decrease from the first to the last cue bin (see Figure 5L, Table S6), unlike all other groups, and did freeze less than *Pten*^+/+^ males in response to the first cue presentation (see Figure S6F, Table S6). Additional minor differences were found: *Pten*^+/-^ females froze significantly longer during the first bin of extinction trial 2 (see Figure S6G, Table S6), and *Pten*^+/-^ males had a trend for freezing less overall (p = 0.070) and during the fourth bin of extinction trial 1 (p = 0.086; see Figure S6H, Table S6).

Taken together, these results show that female *Pten*^+/-^ mice have predominantly intact fear conditioning, regardless of the strength of the training or interval between training and test, as well as largely normal cued fear conditioning extinction. However, male *Pten*^+/-^ mice may have slight impairments in remote memory for fear conditioning and cued fear conditioning extinction.

#### Pattern of behavior and brain overgrowth

To gain insight into the association between brain overgrowth and behavioral abnormalities, we used the existing literature to map behaviors onto brain regions (see Figure S7) in order to examine how altered brain region volume and abnormal behavior may coincide. In addition to the behavioral results presented in this paper (Figures 2, 3, 4, 5, and S2–S6), we also included the outcomes of all behavioral assays performed on the *Pten*^*tm1Rps*^ germline haploinsufficient mouse line to date (Clipperton-Allen and Page, 2014, 2015; Huang et al., 2016; Page et al., 2009a; Sejourne et al., 2015), and grouped them into behaviors that did and did not show behavioral phenotypes. We then used our new analysis, the relative deviation from *Pten*^+/+^ growth trajectory (Figures 1C and 1E), as well as the previously published relative volume differences from the MRI analysis (Clipperton-Allen et al., 2019) to determine significantly altered brain regions (red, relative volume increase; blue, relative volume decrease; see Figure S7). We found that in general, behaviors associated with relatively increased brain regions were more likely to show phenotypes. Behaviors associated with unchanged or relatively decreased regions were less consistent, with some showing phenotypes (e.g., NOR) and others not (e.g., SSRT; Figure S7). Thus, we can predict that behaviors associated with relatively enlarged brain regions are likely to show phenotypes, while unchanged or relatively decreased brain regions have little predictive value.

### Developmental timecourse suggests sensory abnormalities precede social deficits

The most consistent phenotypes we have identified in *Pten*^+/-^ mice are in the sensory (AST and ASH/PPI) and social (social approach in females and social recognition in males) domains. Interestingly, brain regions associated with these behaviors have relatively increased volume in adults (Clipperton-Allen et al., 2019) and show relatively increased growth trajectories (e.g., sensory: pons, inferior colliculus, superior colliculus; social: hypothalamus and medial septum; see Figures 1E, S7), but are not significantly enlarged in P7 *Pten*^+/-^ brains (Clipperton-Allen et al., 2019). To explore the development of these abnormal behaviors, we tested juvenile (P21–25), adolescent (P35 and P45), and adult (>P56) mice on the AST and 3-chamber social approach assays.

#### Acoustic startle threshold test

We largely replicated the high dB hyporeactivity observed in adults of both sexes (see Figures 2C, 2E, and 2K–2L, Figures 6C(ii), 6F(ii), 6I(iii), and 6L(iii), Figures S2A, S2B, S2G, and S2I, Figures S8C, S8I, S8O(iii), and S8R(iii), Tables S3 and S7), and the low dB hypersensitivity in females (Figures 2B and 2G, Figures 6C(i) and 6I(i), Figures S2A and S2G, Figures S8C and S8O(iii), Tables S3 and S7), but found developmental sexual dimorphism. When we looked at the low-dB module (0–25 dB above background), we found that juvenile *Pten*^+/-^ females showed increased startle amplitude at 15 dB above background, with a trend to higher startle amplitude at 20 dB above background (p = 0.091), and a lower startle threshold (first significant startle response at 15 dB above background), unlike *Pten*^+/+^ females, which significantly startled to stimuli of 25 dB above background and higher (see Figure 6A(i), Table S7). Similarly, adolescent *Pten*^+/+^ females showed a trend to startle at 20 dB above background (p = 0.072), while *Pten*^+/-^ females trended toward startling at 15 dB above background (p = 0.060; see Figure 6B(i), Table S7). However, it should be noted that these differences were not present in juveniles or adolescents when we analyzed the log-normal startle amplitude (see Figures S8A and S8B, Table S7). This cohort of adult females did not show a lower threshold in *Pten*^+/-^ mice, as both genotypes startled to 10 dB above background, although the *Pten*^+/-^ females did have a higher startle amplitude at 25 dB above background, with a trend at 30 dB above background (p = 0.068; see Figure 6C, Table S7). However, when we looked at the high-dB module, we found that only the adult *Pten*^+/-^ females showed the hyporeactivity phenotype, with a main effect of genotype and significantly lower startle amplitude than *Pten*^+/+^ females to stimuli of high intensity (see Figures 6A(ii), 6B(ii), and 6C(ii), Table S7). Female mice of all ages showed significant startle responses to stimuli of 25 dB above background or higher (see Figures 6A–6C, Table S7).

The results from fitting sigmoid curves to the female data (see Figures 6G–6I, Figures S8M–S8O) were similar to the more traditional analysis. Adult females showed the same pattern as before, with decreased 5% startle threshold (see Figure 6I(i), Table S7) and maximum startle amplitude (see Figure 6I(iii), Table S7), but no differences in midpoint, slope, or model fitting error (Figure 6I(ii,iv), Figure S8F, Table S7). However, no genotype differences were found on any sigmoid measure for juvenile females (see Figure 6G, Figure S8D, Table S7), with only a trend to decreased sigmoid slope in the adolescents (p = 0.059; Figure 6H(i-iv), Figure S8E, Table S7).

Male mice showed a different pattern of phenotype development. Only the adult males showed a genotype difference for the low-dB module (see Figure 6D(i), 6E(i), 6F(i), Figures S8G–S8I, Table S7), with *Pten*^+/-^ mice showing increased startle amplitude at 20 dB above background (as well as to the 0 dB above background “stimulus”; see Figure 6F(i), Table S7), although these differences were not present in the log-normal startle data (see Figure S8I, Table S7). However, in P45 and this cohort of adult males, the *Pten*^+/+^ mice actually had a lower startle threshold than *Pten*^+/-^ mice (see Figures 6E(i), 6F(i), Table S7). The startle threshold for P21 males was somewhat unclear, as while the lowest stimulus to which juvenile *Pten*^+/-^ males startled (5 dB above background) was lower than that of *Pten*^+/+^ males (15 dB above background), neither genotype startled to the 10 dB above background stimulus, both startled to stimuli of 20 dB above background and higher, and there were no genotype differences in the magnitude of the startle response in the low-dB module, except for the 0 dB above background “stimulus” (see Figure 6D(i), Table S7).

In the high-dB module, however, males of all three ages showed main effects of genotype, with decreased startle amplitude to higher-intensity stimuli (albeit with trends at 40 dB above background for P45, p = 0.070, and at 50 dB above background for adults, p = 0.094; see Figures 6D(ii), 6E(ii), 6F(ii), Table S7); juveniles were also hyporeactive to the 35 dB above background stimulus (see Figure 6D(ii), Table S7). Similar results were found for the log-normal startle data (see Figures S8G–S8I, Table S7). All males significantly startled to stimuli of 30 dB or more above background (see Figures 6J–6L, Table S7).

Sigmoid fitting to the data produced consistent results (see Figures S8P–S8R): all three age groups of male *Pten*^+/-^ mice showed decreased maximum startle amplitudes (Figures 6J(iii), 6K(iii), 6L(iii), Table S7), but no differences on any other sigmoid measures (see Figure 6J–6L, Figures S8J–S8L, Table S7).

These data indicate that female *Pten*^+/-^ mice show some evidence of hypersensitivity to low dB stimuli as juveniles and adults, but only show hyporeactivity to high dB stimuli in adulthood (see Table 1). Male *Pten*^+/-^ mice, however, show hyporeactivity to high dB stimuli throughout development.

**Table 1:** Summary of developmental timecourse of behavioral deficits

Test	Phenotype	Juv.^a^	P35^b^	P45^c^	Adult^d^
**Females**					

Acoustic Startle Threshold	Hypersensitive at Low dB	Yes	n/a	No	Yes
	Hyporesponsive at High dB	No	n/a	No	Yes
Three-Chamber Social Approach	Impaired Social Preference	No	No	Yes	Yes

**Males**					

Acoustic Startle Threshold	Hypersensitive at Low dB	No	n/a	No	Unclear
	Hyporesponsive at High dB	Yes	n/a	Yes	Yes
Three-Chamber Social Approach	Impaired Social Preference	No	No	No	No

a Juv., juveniles (postnatal day 21–25).

b P35, postnatal day 35.

c P45, postnatal day 45.

d Adult, older than postnatal day 56.

#### Three-chamber social approach

The cascading effects theory suggests that differences in sensory processing and responsiveness may precede, and lead to, social deficits and impairments in other higher-level functions in individuals with ASD (e.g., Baranek et al., 2013; Baranek et al., 2018). Thus, we wanted to explore the developmental timecourse of social approach deficits to test this theory in our animal model of macrocephaly/autism syndrome. Adult *Pten*^+/-^ female mice show a consistent, if modest, impairment in social approach behavior, failing to show a preference for a chamber containing a social stimulus in a tube (“mouse + tube chamber”) relative to a chamber containing an empty tube (“empty tube chamber”) across multiple cohorts, experimenters, and locations (e.g., Clipperton-Allen and Page, 2014; Huang et al., 2016; Page et al., 2009a; Sejourne et al., 2015).

**Figure 7:**
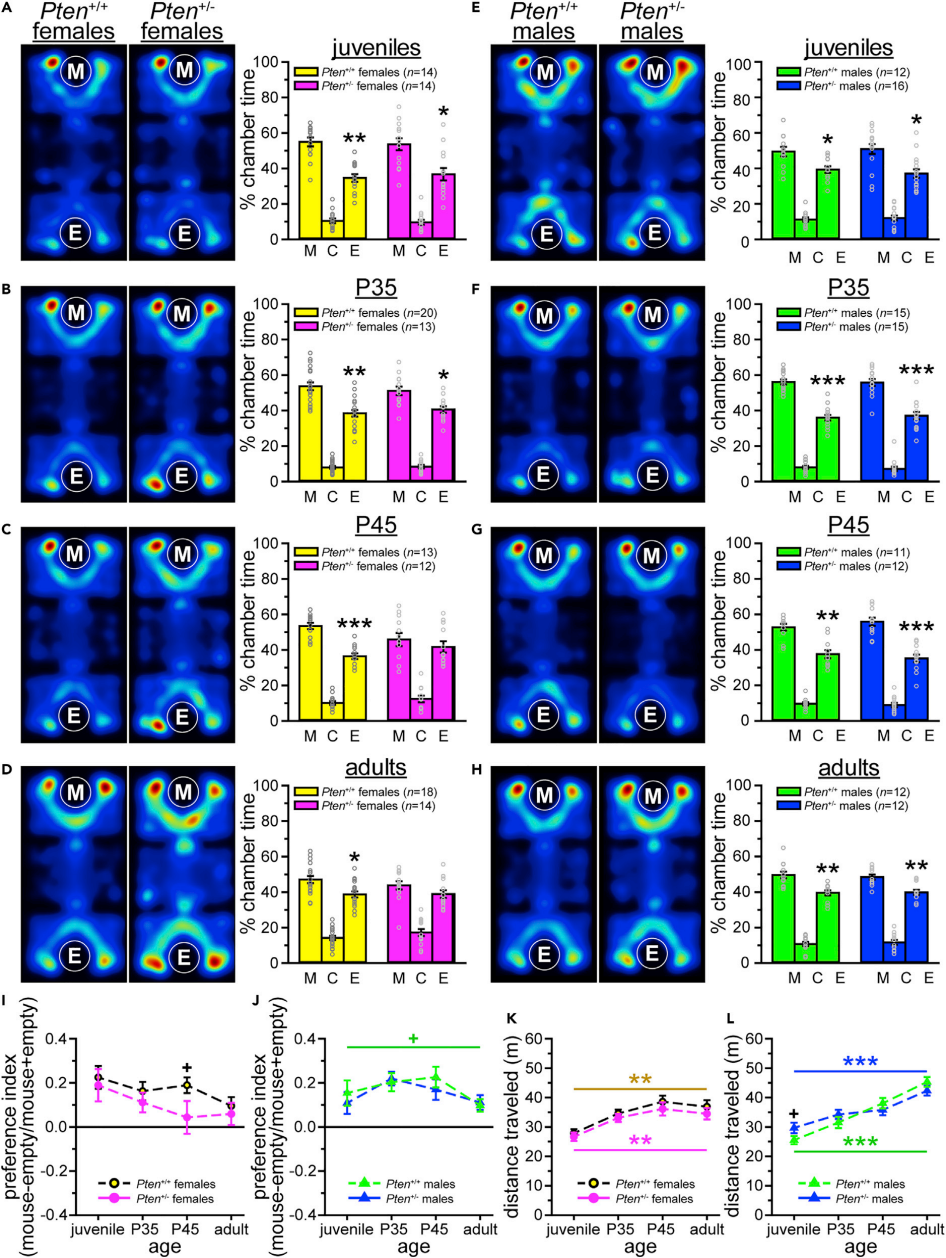
Female *Pten*^+/-^ mice show social deficits from P45, while males show normal social approach behavior throughout development (A–H) All groups show significant social preferences as juveniles (females, A; males, E) and at postnatal day 35 (P35; females, B; males, F). Male mice of both genotypes also show significant social preferences at P45 (G) and in adulthood (H). Female *Pten*^+/-^, but not *Pten*^+/+^, mice fail to prefer the social chamber at P45 (C) and in adulthood (D). (I and J) The preference index, which assesses the strength of the preference for the social chamber [(mouse + tube – empty tube)/(mouse + tube + empty tube) x 100], only differed in the females at P45, with a decrease in the *Pten*^+/-^ mice (I). (K and L) Other than a trend to increased distance traveled in the juvenile *Pten*^+/-^ males (L), no genotype differences were found in locomotion, although all groups showed increased distance traveled across time (females, K; males, L). M, chamber containing mouse + tube; C, center chamber; E, chamber containing empty tube. Heatmaps in A–H are averaged across groups. Data are represented as mean ± SEM. Black symbols, significant preference (paired-samples *t*-tests between mouse + tube and empty tube chambers, A–H) or independent-samples *t*-tests between genotypes (I-L). Colored symbols, effect of age (one-way within-subjects ANOVAs). ∗∗∗ p < 0.001, ∗∗ p < 0.01, ∗ p < 0.05, + p < 0.10. See also Table S7.

The social approach test produces a dichotomous variable, such that mice either do or do not have a social preference, defined as spending more time in the mouse + tube chamber than the empty tube chamber. Social preferences were present in all males (see Figures 7E–7H, Table S7), all female *Pten*^+/+^ mice (see Figures 7A–7D, Table S7), and juvenile and P35 *Pten*^+/-^ females, but not in *Pten*^+/-^ females at P45 or adulthood (see Figures 7A–7D, Table S7). Additionally, we employed a preference index [(time in mouse + tube chamber – time in empty tube chamber)/(time in mouse + tube chamber + time in empty tube chamber)] as a measure of the magnitude of difference in time spent between social and non-social chambers (i.e., the strength of the preference), distinct from the presence or absence of a social preference. Only the P45 females showed a trend to a genotype difference on the preference index (p = 0.080; see Figures 7I and 7J, Table S7), and no genotype differences were found in locomotion, except for a trend in juvenile males (p = 0.076,Figures 7K and 7L, Table S7). All groups showed an increase in locomotion over time (see Figures 7K and 7L, Table S7), but only *Pten*^+/+^ males showed a trend to a change in preference index over time (p = 0.086; see Figure 7J, Table S7).

These data show that the social approach deficit (specifically, a lack of social preference) that we have previously observed in females (e.g., Clipperton-Allen and Page, 2014; Huang et al., 2016; Page et al., 2009a; Page et al., 2009b; Sejourne et al., 2015) is not present at weaning, but develops between P35 and P45 (see Table 1). As we have shown in some (Page et al., 2009a) but not all (Clipperton-Allen and Page, 2014) cohorts, *Pten*^+/-^ males did not have social approach deficits in adulthood, and we show here that this is consistent throughout development.

## Discussion

### Overgrown brain regions predict alterations in associated behaviors

Using the dataset we previously acquired via MRI (Clipperton-Allen et al., 2019), we performed new analyses to identify brain regions showing altered growth trajectories, resulting in larger absolute and altered relative (percent of total brain) volume. As we have previously found a limited number of consistent, replicable behavioral phenotypes in *Pten*^+/-^ mice (i.e., social approach deficits in females, social recognition deficits in males, and PPI in both sexes; Clipperton-Allen and Page, 2014; Clipperton-Allen and Page, 2015; Clipperton-Allen and Page, 2020; Huang et al., 2016; Page et al., 2009a; Sejourne et al., 2015), we used these abnormally scaled regions as an unbiased screen to select additional behavioral assays (associated with these areas) to test. We found *Pten*^+/-^ phenotypes in the majority of behaviors associated with relative (percent of brain volume) increases in volume and growth trajectory, whereas only some of the behaviors associated with brain regions with relative decreases or no change were altered in *Pten*^+/-^ mice (see Figure S7). The latter included a phenotype on a cognitive task in females, associated with relatively decreased (PFC, ACC in the frontal lobe, and perirhinal cortex in the parieto-temporal lobe) and unchanged (entorhinal cortex and hippocampus) regions (Figure S7; Cohen and Stackman, 2015; Warburton and Brown, 2015; Weible et al., 2009). *Pten*^+/-^ females showed no preference for a novel object, which is interesting given that they have previously been shown to have normal social recognition in a habituation/dishabituation task (Clipperton-Allen and Page, 2014). This deficit may be related to a slight spatial memory impairment, as it has been suggested that the NOR assay has a spatial component (Forwood et al., 2005), whereas the stimulus mice were placed in the same location during the habituation/dishabituation task (Clipperton-Allen and Page, 2014). Consistently, the MWM results suggest a subtle impairment in spatial memory in the female *Pten*^+/-^ mice. However, *Pten*^+/-^ females show superior performance in the tissue condition of the puzzle box assay, which is associated with regions with relatively increased (hypothalamus), decreased (ACC and PFC in the frontal lobe), and unchanged (hippocampus and thalamus) growth trajectories and/or volume (Ben Abdallah et al., 2011; O'Connor et al., 2014). This suggests that these mice may have enhanced cognitive function or flexibility. One caveat for the brain volume/behavior associations is that the MRI assay was exclusively performed on male mice; thus, sexual dimorphism in the abnormal growth trajectories and resulting abnormal brain scaling could account for the lack of predictive value of decreased or unchanged relative volume and/or growth trajectory for brain regions in these assays.

### Brain regions with the largest overgrowth are associated with sensory processing, the only behavior abnormal in *Pten*^+/-^ mice of both sexes.

The most overgrown regions (e.g., pons, inferior colliculus, and medulla) were those involved in sensory processing, particularly acoustic startle threshold, habituation, and PPI assays (Carlson and Willott, 1998; Fendt et al., 2001; Koch and Schnitzler, 1997; Koch, 1999; Yeomans and Frankland, 1995). These behaviors not only showed the strongest and most consistent phenotypes, and were the only behavioral phenotypes observed in *Pten*^+/-^ mice of both sexes (Clipperton-Allen and Page, 2020), but they are also of particular interest due to sensory abnormalities being a key ASD symptom. Our findings indicated that while *Pten*^+/-^ females are hypersensitive to low-intensity stimuli, *Pten*^+/-^ mice of both sexes are hyporesponsive to high-intensity stimuli. This is consistent with the behavioral abnormalities observed in humans with *PTEN* mutations, who are also hyporesponsive and have deficits in acoustic processing (Busch et al., 2019).

As this was one of the most consistent and strongest phenotypes we have observed in *Pten*^+/-^ mice, we tested mice at different ages to establish a developmental timecourse of the acoustic startle phenotype(s). We found that male and female *Pten*^+/-^ juveniles displayed different aspects of the adult startle phenotype, with juvenile females showing hypersensitivity and a lower startle threshold but no hyporeactivity, and juvenile males being hyporeactive to high-intensity stimuli. When we performed a similar analysis of social approach deficits in the three-chamber assay, we found that the lack of social preference only emerged at P45 in *Pten*^+/-^ females, while males remained normal. Thus, our data suggest that sensory abnormalities may precede social impairments in *Pten*^+/-^ mice (summarized in Table 1). Similar observations have been made in humans with ASD, leading to the so-called cascading effects theory, which proposes that early differences in sensory responsiveness and processing may lead to cascading effects on higher-level function, including social skill (Baranek et al., 2013, 2018). While not specifically in individuals with ASD and *PTEN* mutations, and not associated with brain scaling abnormalities, there is some empirical evidence to support this theory. For example, both hyper- and hyporesponsiveness to sensory stimuli have been linked to social and communication deficits (Baranek et al., 2013; Ben-Sasson et al., 2009), and increased sensory seeking in the late second year of life is more common in individuals later diagnosed with ASD (Baranek et al., 2018). Further research is necessary to determine whether the male juvenile hyporeactivity precedes the emergence of the social recognition deficit observed in *Pten*^+/-^ male mice (Clipperton-Allen and Page, 2014), as *Pten*^+/-^ males do not show a consistent social approach phenotype (Clipperton-Allen and Page, 2014; Page et al., 2009a).

Interestingly, this pattern shows a striking resemblance to that seen in *Fmr1* knockout (KO) mice. Like *Pten, Fmr1* is a high-risk ASD gene and a negative regulator of the PI3K-Akt-mTOR pathway, and hemizygous (*Fmr1*^*-/y*^) male mice have similar patterns of transient developmental phospho-S6 increases and cellular hypertrophy in a subset of neurons to *Pten*^+/-^ mice (Huang et al., 2016). *Fmr1*^*-/y*^ males on a C57BL/6J background show increased startle response amplitude to low-intensity stimuli, and decreased responding to high-intensity stimuli (Errijgers et al., 2008; Nielsen et al., 2002). Although there have been very few examinations of female *Fmr1* heterozygous (*Fmr1*^+/−^) or KO (*Fmr1*^−/−^) mice, one study on the C57BL/6J background found no effect of genotype on acoustic startle in either male or female *Fmr1* mutants (Ding et al., 2014), while examination of both sexes on the FVB background found reduced startle in *Fmr1*^*-/y*^ males, but no difference between wild-type (WT), *Fmr1*^+/−^, and *Fmr1*^−/−^ females (Kokash et al., 2019). Similarly, inconsistent results have been found for sensorimotor gating in *Fmr1*^−/−^ mice (de Vrij et al., 2008; Frankland et al., 2004), although an alternative approach to analyzing the effects of PPI suggests that there are no effects of *Fmr1* KO in rats (Miller et al., 2020). Thus, it is unclear whether the PPI phenotype present in *Pten*^+/-^ mice is similar to that of *Fmr1* mutant mice. While not on the C57BL/6J background, two studies have investigated the developmental timecourse of AST or ASH/PPI in male *Fmr1*^*-/y*^ mice on the FVB background. Both studies found decreased startle response amplitude in juveniles, like in our *Pten*^+/-^ juvenile males, but either reduced (Yun et al., 2006) or normal (Kokash et al., 2019) baseline startle in adults. In the ASH/PPI study, PPI was decreased in *Fmr1*^*-/y*^ mice at both juvenile and adult ages (Kokash et al., 2019).

While we cannot draw any specific conclusions about the mechanism of the acoustic startle abnormalities observed in *Pten*^+/-^ mice, several possible explanations exist. One possibility is that this may be due to hyperconnectivity or hyperexcitability in the acoustic startle circuitry. We have previously shown that the mPFC-BLA circuit is hyperconnected in *Pten*^+/-^ mice (Huang et al., 2016), and the increased size, not only in absolute but also in relative terms, as well as the increased growth trajectory, of brain regions key for acoustic startle responses (e.g., pons, IC, etc.) might point to hyperconnectivity. This could be due to reduced pruning in the *Pten*^+/-^ mice, resulting in the altered velocity of growth; decreased autophagy and pruning have been shown in response to increased mTOR signaling in mice with a mutation in *Tsc*, another negative regulator of the PI3K-Akt-mTOR pathway (Tang et al., 2014). Consistently, *Pten*^+/-^ mice show abnormal pruning due to altered microglia at two but not six weeks of age (Xu et al., 2020). Future research will attempt to isolate the mechanism responsible for the *Pten*^+/-^ sensory processing phenotype, and to elucidate the link between abnormal brain scaling and behavioral phenotypes.

### Conclusions

In these experiments, we sought to understand how MRI data can inform hypotheses about behavioral phenotypes and guide the selection of those behavioral assays most likely to show abnormal responses. We extended the analysis of our MRI dataset to determine which brain regions deviated the most from expected developmental trajectories. Using these data, we found a strong sensory processing phenotype associated with the most enlarged brain regions. While in adulthood, hyporesponsiveness was present in both sexes (with hypersensitivity in females), an interesting sexual dimorphism emerged in juveniles, with *Pten*^+/-^ females showing hypersensitivity (lower startle threshold, increased startle amplitude to low-dB stimuli) and *Pten*^+/-^ males being hyporesponsive to high-intensity stimuli. We also determined that our previously reported deficit in social approach behavior in *Pten*^+/-^ females emerges in adolescence (between P35 and P45). We found that increases in brain region volume and growth trajectory, relative to overall brain size, have good predictive value, such that in a mouse model of mutations in the ASD risk gene *PTEN*, behaviors associated with enlarged regions are typically also abnormal. Unchanged or relatively decreased regions fail to have predictive value.

### Limitations of the study

Our results suggest that behaviors associated with brain regions that are enlarged or show accelerated growth trajectories above total brain volume enlargement in *Pten*^+/-^ mice are likely to show abnormal results. However, there are some caveats to this interpretation. First, the MRI was only performed on male mice, whereas the behavior tests were all done in both sexes. However, since the magnitude of brain enlargement is similar in males and females, we believe that the pattern of abnormal brain scaling is also likely similar. We also focus exclusively on the volumetric analysis provided by MRI. It is possible that other quantitative measures of tissue properties, including relaxation times, fractional anisotropy, and mean diffusivity, could show differences associated with behavioral phenotypes. These are the target of future investigations and will help to refine the association between structural brain abnormalities and abnormal behavior, leading to improved predictive value. Another caveat is that the P7 and P60 MRI were performed in different animals due to the postmortem nature of the imaging. Additionally, the behavioral assays and the MRI analyses were performed on different mice, thus limiting our ability to perform animal-by-animal correlations and find a more direct link between the behavior and brain region abnormalities in our mutant mice. Therefore we are, by necessity, drawing conclusions from the averaged results of multiple animals. Furthermore, the mechanistic link between *Pten* mutations, changes in brain region volume, and behavior remains unresolved, although elucidating this link, based on the groundwork laid by these analyses, is the aim of future studies.

## STAR★Methods

### Key resources table

**Table:** 

REAGENT or RESOURCE	SOURCE	IDENTIFIER
**Chemicals, peptides, and recombinant proteins**		

ProHance (Gadoteridol)	Bracco Diagnostics	Manufacturer #00270111104; CAS 120066-54-8

**Experimental models: Organisms/strains**		

Mouse: *Pten*^+/-^: *B6.129-Pten*^*tm1Rps*^	NCI Frederick	RRID: MGI:2179044
Mouse: *Pten*^+/+:^ C57BL/6J	The Jackson Laboratory	RRID: IMSR_JAX:000664

**Software and algorithms**		

BORIS (Behavioral Observation Research Interactive Software)	Friard and Gamba, 2016	https://github.com/olivierfriard/BORIS
Ethovision XT	Noldus Information Technology	RRID:SCR_000441
SR-LAB	San Diego Instruments	https://sandiegoinstruments.com/product/sr-lab-startle-response/
PASW 18 (IBM SPSS Statistics)	IBM Corporation	RRID:SCR_019096

**Other**		

Organic Whole Millet (seed rewards)	Arrowhead Mills	Item model # 0009800007813

### Resource availability

#### Lead contact

Further information and requests for resources and reagents should be directed to and will be fulfilled by the lead contact, Damon T. Page (paged@scripps.edu).

#### Materials availability

This study did not generate new unique reagents.

### Experimental model and subject details

#### Mouse models

Mice of the *B6.129-Pten*^*tm1Rps*^ line (RRID: MGI:2179044; Podsypanina et al., 1999) were obtained from the repository at the National Cancer Institute at Frederick, where they were already backcrossed onto a congenic C57BL/6J background by the Donating Investigator. The line has been maintained by backcrossing to C57BL/6J mice (RRID: IMSR_JAX:000664; The Jackson Laboratory strain #000664) for more than 10 generations. Mice used in this study were generated by crossing *Pten*^*tm1Rps/+*^ (*Pten*^+/-^) male mice with wild-type (*Pten*^+/+^) females. After weaning, mice were held on ventilated racks (Model No. MD75JU160MVPSHR, Allentown Inc., Allentown, NJ, USA) in clear polyethylene cages (19.1 × 29.2 × 12.7 cm^3^; Allentown Inc.) in groups of 3-5, and provided with *ad libitum* food (Teklad Global 18% Protein Extruded Rodent Diet 2920X, Harlan Laboratories, Indianapolis, IN, USA) and water, ¼” corncob bedding, and nestlets.

For the magnetic resonance imaging (MRI), *Pten*^+/-^ males and *Pten*^+/+^ littermate controls were used at either postnatal day 7 (P7) or P60. For behavioral assays, *Pten*^+/-^ mice and littermate *Pten*^+/+^ controls were tested in adulthood unless otherwise specified (see Table S8 for ages). For some tests, one or more cohorts were tested in batteries, which had at least 3-7 days between tests. Test order was selected to minimize carryover effects, and particularly aversive assays (i.e., fear conditioning, Morris water maze) were the final test if part of a battery (see Table S8).

All research was approved by The Scripps Research Institute’s Institutional Animal Care and Use Committee and conducted in accordance with National Institutes of Health and Association for Assessment and Accreditation of Laboratory Animal Care International (AAALAC) guidelines.

### Method details

#### Magnetic Resonance Imaging

##### Brain collection

On postnatal day 7 (P7) or P60, male *Pten*^+/+^ (P7: n = 10; P60: n = 10) and *Pten*^+/-^ (P7: n = 10; P60: n = 9) littermates from seven (P7) or six (P60) litters were collected as previously described (Clipperton-Allen et al., 2019). Briefly, anesthetized mice were perfused with 1X PBS (Life Technologies, Carlsbad, CA, USA) with 10U/ml heparin (Sigma-Aldrich, St. Louis, MO, USA) and 2 mM Pro-Hance (Bracco Diagnostics, Monroe Twp, NJ, USA), followed by 4% paraformaldehyde (PFA; Sigma-Aldrich) containing 2 mM Pro-Hance, then decapitated and the skin, lower jaw, ears, and cartilaginous nose tip were removed. After overnight incubation in the PFA solution, brains were stored in 1X PBS with 2 mM Pro-Hance and 0.02% sodium azide (Sigma-Aldrich) until imaged.

##### Imaging, registration, and analysis

Images were acquired on a 7.0 Tesla MRI scanner (Agilent Inc., Palo Alto, CA, USA) (Nieman et al., 2005, 2006), and images were linearly and non-linearly registered toward a pre-existing atlas, transformed, and averaged to calculate the volume of individual gray matter, white matter, and ventricular structures as previously described (Clipperton-Allen et al., 2019). Acquisition and registration specifics are detailed below. Relative volume was calculated as [(brain region volume)/(whole brain volume) x 100]. The main findings of these experiments are presented in a previous paper (Clipperton-Allen et al., 2019).

P7 Image Acquisition. The contrast required for registration and assessment of volume is not acceptable with our typical T2-weighted imaging sequence. Therefore, diffusion weighted imaging was performed to enhance the contrast between white and gray matter to aid in the registration and volume measurements.

P7 Diffusion Imaging Sequence. The diffusion sequence uses an in-house custom built 16-coil solenoid array to acquire images from 16 brains in parallel (Nieman et al., 2007). The diffusion sequence used was a 3D diffusion-weighted FSE, with TR = 270 ms, echo train length = 6, first TE = 30 ms, TE = 10 ms for the remaining 5 echoes, one average, FOV = 25 × 14 × 14 mm, and a matrix size of 450 × 250 × 250, which yielded an image with 56 μm isotropic voxels. One b = 0 s/mm^2^ image was acquired and 6 high b-value (b = 2147 s/mm^2^) images were acquired at the following directions (1,1,0), (1,0,1), (0,1,1), (−1,1,0), (−1,0,1) and (0,1,-1) corresponding to (Gx,Gy,Gz). Total imaging time was ∼14 h.

P60 Anatomical Imaging Sequence. To detect volumetric changes in the older animals, we used a T2-weighted, 3D fast spin-echo sequence, with a cylindrical acquisition of k-space, a TR of 350 ms, and TEs of 12 ms per echo for 6 echoes, field-of-view equaled to 20 x 20 x 25 mm^3^ and matrix size equaled to 504 x 504 x 630. Our parameters output an image with 0.040 mm isotropic voxels. The total imaging time was ∼14 h (Spencer Noakes et al., 2017).

Registration and Analysis. To visualize and compare the mouse brains the images were registered together, for the P7 Images the 6 high b-value images were averaged together to make a high contrast image necessary for accurate registration. For the P60 images the acquisition images were used. These images, in two separate pipelines, were linearly (6 parameter followed by a 12 parameter) and non-linearly registered together. All scans were then resampled with the appropriate transform and averaged to create a population atlas representing the average anatomy of the study sample. All registrations were performed using a combination of the mni_autoreg tools (Collins et al., 1994) and ANTS (Avants et al., 2011). The result of the registration was to have all scans deformed into exact alignment with each other in an unbiased fashion. For the volume measurements, this allowed for the analysis of the deformations needed to take each individual mouse’s anatomy into this final atlas space, the goal being to model how the deformation fields relate to genotype (Lau et al., 2008; Nieman et al., 2006). The Jacobian determinants of the deformation fields are then calculated as measures of volume at each voxel. These measurements can be examined on both a regional and a voxel-wise basis in order to localize the differences found within regions or across the brain. Multiple comparisons were controlled for by using the False Discovery Rate (FDR; Genovese et al., 2002).

##### Additional analyses

We extended the analysis of the data from our MRI study (Clipperton-Allen et al., 2019) to determine how the developmental trajectory is altered in the individual brain regions of *Pten*^+/-^ mice. Two developmental trajectory measures were calculated on the absolute and relative volumes of each brain region:

1) Growth index, calculated separately for each brain region and genotype as:

$$Pten^{+/+}:\;\frac{\left(P60\;value_{\text{individual }{\mathrm{Pten}}^{+/+}\text{mouse}}-\;average\;P7\;value_{{\mathrm{Pten}}^{+/+}\text{mice}}\right)}{average\;P7\;value_{{\mathrm{Pten}}^{+/+}\text{mice}}}$$

$$Pten^{+/-}:\;\frac{\left(P60\;value_{\text{individual }{\mathrm{Pten}}^{+/-}\text{mouse}}-\;average\;P7\;value_{Pten^{+/-}\text{mice}}\right)}{average\;P7\;value_{{\mathrm{Pten}}^{+/-}\text{mice}}}$$

*2) Pten*^+/-^ % deviation from *Pten*^+/+^ growth trajectory *x*, calculated for each brain region (see Figures 1B–1E):

$$x=\frac{y-\hat{y}}{\hat{y}}\;\times 100$$

where

$$y\;=\mathrm{region}\;\mathrm{size}\;\mathrm{for}\;\mathrm{individual}\;P60\;Pten^{+/-}\;\mathrm{mouse}\;\hat{y}\;=\mathrm{predicted}\;\mathrm{region}\;\mathrm{size}\;\mathrm{for}\;\mathrm{average}\;P60\;{\mathrm{Pten}}^{+/-}\;\mathrm{mouse}\;\;={\text{mean}}_{\text{P}7\;{\mathrm{Pten}}^{+/-}\;\text{mice}}\times w\;\;w=Pten^{+/+}\;\text{growth trajectory}=\;=\frac{{\text{mean}}_{P60\;{\mathrm{Pten}}^{+/+}\;\text{mice}}}{{\text{mean}}_{P7\;{\mathrm{Pten}}^{+/+}\text{ mice}}}$$

The growth indices (calculation 1) for each brain region were correlated within each genotype, producing four correlation matrices (absolute volume in *Pten*^+/+^, absolute volume in *Pten*^+/-^, relative volume in *Pten*^+/+^, relative volume in *Pten*^+/-^). We then assessed the difference in significant correlations between genotypes, presented in absolute (Figures 1A, S1A, Table S1) and relative (Figure S1B, Table S1) matrices of the difference in brain region growth index correlations between genotypes.

#### Behavioral assays

##### General procedures

All behavior tests were performed on both male and female mice, and the sexes were analyzed separately, as sexual dimorphism is common in mice with *Pten* mutations (Clipperton-Allen and Page, 2014, 2020). Adult behavior tests were performed during the dark (active) phase of the reversed light-dark cycle under red light, unless otherwise specified. Mice were moved into the testing area at least 1 hour prior to testing. Apparati were cleaned with 70% ethanol (EtOH; Sigma-Aldrich), 1% Micro-90 (International Products Corporation, Burlington, NJ, USA) and/or quatricide (2 oz/gallon; Pharmacal Research Laboratories, Inc., Waterbury, CT, USA), unless otherwise stated. Manual scoring was performed by a trained observer blind to sex and genotype using a stopwatch (Puzzle Box) or BORIS [(Friard and Gamba, 2016); Novel Object Recognition (NOR), Single-Seed Reaching Task (SSRT)]. Automatic scoring was performed using the Ethovision XT video tracking system [Morris Water Maze (MWM), Weak (WFC) and Remote Memory for (RMFC) Trace Fear Conditioning, Cued Fear Conditioning Extinction (FCExt), Open Field Test (OFT); RRID:SCR_000441; Noldus Information Technology, Wageningen, The Netherlands] or assay-specific software [Acoustic Startle Threshold (AST), Acoustic Startle Habituation (ASH), Pre-pulse Inhibition (PPI), Rotarod Learning]. Mice underwent 1-4 tests, spaced at least 3–7 days apart (see Table S8). Most assays were tested in at least two cohorts, which were combined if results were the same (Table S8). If two cohorts differed, a third cohort was run to confirm the results. No cohorts were excluded from analysis. Details of these paradigms are listed below.

##### Acoustic startle threshold test

For the adult analysis, mice were tested during the dark phase with red room lights. For the timecourse analyses, mice were tested during the light phase with dim white room lights. Each mouse was placed inside a clear acrylic tube (P21: 28 mm inner diameter, 90 mm long; P45 and adult: 39 mm inner diameter, 128 mm long) secured to a platform with a piezoelectric accelerometer attached beneath the tube inside a ventilated, sound-attenuating chamber with no house or cue lights on (San Diego Instruments, San Diego, CA, USA). Following a 5 min acclimation period, mice received trials of 40 ms white noise stimuli of varying intensities (from 0-50 dB above the 70 dB background white noise by 5 dB increments). Stimuli were presented in a pseudorandom order, with 8 presentations per intensity, plus 8 control trials (no stimulus), and variable 8-23 s inter-trial intervals. The maximum whole-body flinch response to each stimulus (“startle response amplitude”) was recorded using SR-Lab software (San Diego Instruments), which takes 65 consecutive 1 ms readings from the beginning of stimulus onset. Typically, the startle magnitude will increase with increasing stimulus dB (see Figure 2A), and the acoustic startle threshold was defined as the lowest dB at which a significant startle is observed. Startle amplitude (both raw and log-normalized) for each type of stimulus was averaged across the 8 presentations.

In addition to the traditional analyses of startle amplitude, we also fitted sigmoid functions to the log-normal data as per Miller et al., (2020), using the following formula:

$$N\left(\text{s}\right)=\frac{m_{\text{max}}}{1+e^{-r\left(s-s_{0}\right)}}$$

where *s* is the startle sound level, *m*_max_ is the saturation point (maximal movement to a startling sound, as extrapolated from the sigmoid function), *s*_0_ is the sound that produces a startle at 50% of saturation, and *r* is the slope of the sigmoid (the rate at which the startle response changes from zero to saturation).

Sigmoid functions were fit to the data for each mouse using the Python code provided by Miller et al. (2020). As this code was produced with the intent of providing a better analysis of pre-pulse inhibition, it required pre-pulse sound levels and delays for each mouse; these were all set at 0. This analysis produced the 5% startle threshold (stimulus that produces a startle amplitude of 5% of saturation), saturation, midpoint, and sigmoid slope measures, as well as the total root mean squared error (RMSE) between the data and the model (“model fitting error”). It should be noted that our highest startle sound, 50 dB above background, may be lower than is necessary to reach true saturation of the startle response; thus, we calculated the maximum startle for each genotype by taking the highest average startle amplitude for each mouse (regardless of sound intensity).

##### Acoustic startle habituation and pre-pulse inhibition test

Each mouse was placed in the startle apparatus described above. Following a 5 min acclimation period, mice received trials of white noise stimuli in three phases, with the same background noise and inter-trial intervals as the acoustic startle threshold test above: I) pre-PPI startle, II) mixed startle and PPI, and III) post-PPI startle. Phases I and III consist of 6 startle trials with a 40 ms white noise stimulus at 120 dB. During phase II, mice receive a total of 52 trials of three types, presented in a pseudorandom order: i) 12 startle trials (as in phases I and III), ii) 10 control trials (no stimulus), iii) 10 PPI trials for each pre-pulse stimulus intensity (20 ms pre-pulse stimulus at 4, 8, or 16 dB above background, followed by 120 dB startle stimulus 100 ms after pre-pulse onset). Startle response amplitude for each type of stimulus was averaged across presentations within each phase. Percent PPI was calculated as [(phase II startle response amplitude – pre-pulse startle response amplitude)/(phase II startle response amplitude) x 100]. Startle habituation was calculated as [(phase I startle response amplitude – phase III startle response amplitude)/(phase I startle response amplitude) x 100] for each mouse, and group means were graphed. As some animals had “negative” startle habituation values (i.e., they startled more during phase III than phase I), this results in mean startle habituation scores for groups that are different than one would expect by looking at the difference in the mean phase I response and the mean phase III responses without factoring in the individual animals' values.

##### Novel object recognition test

The NOR apparatus was a white acrylic box (42 × 42 × 42 cm) with removable circular disc cutouts in each corner (95 mm diameter). Objects consisted of plastic hair clips (3 × 3.75 × 3.75 cm) and small wire strainers (6.5 cm diameter, 3 cm high) that were attached to circular discs with white lab tape (see Figure 4A). Objects were removed from the discs, and discs, objects, and the arena were cleaned with quatricide between trials. The test was performed in dim white light conditions.

The test consisted of a habituation day and three trials over the following two days (see Figure 4A). On day 1, each mouse was put into the NOR apparatus with blank (empty) discs in all corners and allowed to explore for 10 min. In Phase 1 (sample 1, day 2), mice were returned to the NOR apparatus for 10 min, which now contained two identical objects placed on the discs in opposing corners (object and corner location were counterbalanced). Phase 2 (sample 2, day 3) was the same as Phase 1, except that the two identical objects were placed in the opposite corners. Four hours later, mice were tested in Phase 3 (test, day 3), in which one of the identical objects was replaced with a novel object, and the objects were placed in the same corners as Phase 1 (see Figure 4A). Objects and corner placements were counterbalanced within and across genotypes and sexes throughout.

Following testing, videos were scored using BORIS to record the time spent investigating each object. Mice were considered to be investigating when their noses were less than 2 cm from the objects, but they were not climbing or sitting on the objects. Two main categories of analysis were performed.

Novel Object Preference. To assess preference for a novel object, we calculated the percent time investigating (each object) as {[(time investigating novel or familiar object)]/[(time investigating novel object) + (time investigating familiar object)] x 100}, and the discrimination index (DI) as {[(time investigating novel object) – (time investigating familiar object)]/[(time investigating novel object) + (time investigating familiar object)] x 100}. Thus, positive DI scores indicate increased time investigating the novel object, and negative scores mean more time spent with the familiar object.

Total Object Investigation. To determine whether there were differences in the amount of time spent investigating the objects during the sample and test phases, we calculated the percent of each trial spent investigating as {[(time investigating novel object) + (time investigating familiar object)]/[trial duration] x 100}.

##### Puzzle box

The puzzle box test was run as described in O'Connor et al. (2014), except that each mouse was given longer to solve each task (see Table S9). For each trial, the mouse was placed in the puzzle box apparatus (30 × 60 × 30 cm) and the time to reach the goal box (separated into a 30 × 20 cm covered chamber by an opaque wall) was recorded. The entry to the goal box was either open or contained one of four obstructions (see Figure 4F), and each condition was tested three times. Mice received three trials a day for 5 consecutive days, with the third trial of a condition always occurring at the beginning of the second day (see Table S9).

Each condition was designed to require a different technique to succeed, thus demanding cognitive flexibility on the part of the mice. Condition 1 simply required entry through the tunnel. To solve Condition 2, digging was required. Condition 3 had two potential strategies: 1) pull the tissue out, or 2) climb over and push the tissue down. Condition 4 was the most challenging, and required the mice to push the foam “plug” into the goal box (although it was also possible to pull the plug out, it took much more time and was almost never completed in the 360 s allowed). The schedule of testing and time allowed for each condition is summarized in Table S9.

The latency to enter the goal box was recorded, and the percent of maximum time allowed was calculated as [(latency to enter)/(maximum time) x 100] in order to compare across conditions with different maximum durations. The percent maximum time to complete each task (percent time to completion) was averaged across the three trials. We also analyzed the percent of mice that successfully completed the task for each trial. In order to control for decreased motivation or for habituation to the arena or testing procedure, we calculated the difference in time to enter the goal box with no obstacles (Condition 0) between the beginning (trial 1, T1) and end of testing (T2) as (C0T1 latency – C0T2 latency).

##### Open field test

To ensure that differences in performance on the Puzzle Box assay were not due to locomotor or anxiety confounds, we tested the mice used on that assay in the OFT. Mice were placed into the center of a brightly lit open field box (43.8 x 43.8 x 32.8 cm) under ∼240 lux for 5 min while automatically recorded. Locomotion was assessed using total distance traveled. Increased anxiety was indicated by increased time in thigmotaxis (time spent near walls and corners), and decreased time in the center of the field.

##### Rotarod learning

Mice were tested on 3 consecutive days, with a final test 7 days later, to assess motor learning. Each day consisted of 3 trials, with at least 1 hour inter-trial intervals, on a 10.5 cm circumference rotating rod that accelerated from 4-40 RPM over 5 min (Med Associates Inc., St. Albans, VT, USA). The latency to fall was recorded, and averaged across each day.

##### Single-seed reaching task

The SSRT was performed as described in (Chen et al., 2014; Xu et al., 2009). Specifically, the apparatus consisted of a clear acrylic box (8 × 15 × 20 cm) with three vertical slits (14 × 0.75 cm) in the front wall – a center slit on the shaping edge, and two slits on the training edge (opposite). The apparatus was equipped with an acrylic platform (88 × 45 × 12 mm) that had an angled channel down the center for shaping on one side, and two small divots for seed placement during training on the other. Mice were trained to reach through the slit to retrieve millet seed rewards (Arrowhead Mills Organic Whole Millet, Amazon.com) under white light conditions in three phases: habituation, shaping, and training. Mice were maintained at 85-90% of free-feeding weight throughout. On each of the 10-13 consecutive days of the experiment, weights were recorded prior to the start of testing, and mice were fed following its completion.

Habituation. On day 1, mice were placed individually into the apparatus in the shaping (single-slit) configuration with no food platform. Thirty millet seeds were placed on the floor, and the mice were given 15 min to habituate to the both the box and the millet seeds. Mice were required to consume at least 1 seed in order to continue, which all did.

Shaping. On day 2, mice were returned to the apparatus in the shaping configuration, and the channel side of the feeding platform was filled with 30 seeds. Mice were given up to 20 min to reach the criteria of 30 attempts, at least 70% of which were with one forelimb. Shaping was repeated until mice reached these criteria on two consecutive days (2-5 trials).

Training. After meeting the shaping criteria, mice began training, which took place on 7 consecutive days with the apparatus in the training configuration. Seeds were placed one-by-one in the divot corresponding to the preferred paw. Each trial continued until the mouse made 30 attempts or 20 min had elapsed. “Attempts” consisted of successes (reached through, picked up seed, and brought to mouth), drops (reached through, picked up seed, but dropped before bringing to mouth), hits (reached through and hit the seed, but did not pick it up), and misses (reached through but did not make contact with the seed). “Reaches” included all attempts, as well as “uncounted” reaches which did not count towards the 30 attempt criterion: contralateral (reached with wrong paw), in vain (reached with no seed in place), unbaited (reached at the unbaited, non-preferred side slot), and tongue (mouse reached out with tongue to pick up seed) reaches. The total number of reaches was counted live, and trials were videotaped and scored with BORIS to analyze the number and types of reaches made. The total number of reaches, the percent of successful attempts (# success/# attempts x 100), and the number of successful attempts per minute were analyzed.

##### Morris water maze

To assess the spatial learning and memory, as well as the perseverance or cognitive flexibility of *Pten*^+/-^ mice, we performed the MWM reversal test as described in (Shahani et al., 2017; Vorhees and Williams, 2006). Mice were tested in a 122 cm-diameter tank filled with 23°C water made opaque by the addition of non-toxic white tempera paint, in which a 10 cm diameter platform was placed. Distal cues were placed around the tank to guide the mice. There were three phases of testing in the MWM: 1) visible platform test (VPT, Day 0); 2) acquisition training (Females: Day 1–8; Males: Day 1–7); and 3) reversal acquisition training (Females: Day 10–15; Males: Day 9–13).

Visible Platform Test. In the VPT, the platform was raised to be visible above the water; this ensured that the mice had no deficits in visual or swimming ability. Mice were placed in the tank at different start locations for each of the 4 trials, and given 60 s to locate the platform. After 15 s on the platform, mice were dried and placed into cages on hot water heating pads for the 20 min inter-trial intervals.

Acquisition Training. Acquisition training was conducted in the same manner as the VPT, except that the platform was approximately 1 cm below the surface of the water. If mice failed to locate the platform within 60 s, they were gently guided to its location. During acquisition, the platform remained in the same position but the start location was pseudorandomly varied. Training continued until mice met the acquisition criteria of each group reaching the platform within 20 s and having a 95% success rate for reaching the platform.

Reversal Acquisition Training. Reversal training began the day after the post-acquisition probe trial. This training was identical to the acquisition training except that the platform was moved into a new position for the duration of the reversal acquisition trials, with the start location again pseudorandomly assigned. The same criteria were also applied.

Probe Trials. To assess reference memory and memory consolidation, probe trials were performed every two days (before the daily training trials), as well as the day after meeting acquisition and reversal criteria [Females: Day 3, 5, 7, 9 (probe only), 12, 14, 16 (probe only); Males: Day 3, 5, 7, 8 (probe only), 11, 13, 14 (probe only)]. During probe trials, mice were placed in the tank for 60 s with no platform present.

Measures. In the VPT, acquisition training, and reversal acquisition training, the latency to find the platform, distance traveled, and percent of successful trials were recorded and averaged across the 4 daily trials for each mouse. During probe trials, measures included those relating to where the platform was normally located (distance to the platform, number of platform crossings, latency to platform crossing), those based on the platform quadrant (% time in platform quadrant, latency to platform quadrant), and the total distance traveled.

##### Fear conditioning

Fear conditioning was tested as described in (Clipperton-Allen and Page, 2014), with modifications outlined in detail below. Mice were trained in Phenotyper chambers (Noldus Information Technology, RRID: SCR_004074, Wageningen, The Netherlands) containing an electrified floor of 32 steel bars, a speaker, and clear walls, placed inside noise-attenuating boxes with white lights and fans on (Med Associates Inc., St. Albans, VT, USA). The assay had four phases: Phase I) Training consisted of a 2 min baseline followed by presentation(s) of the conditioned (CS, a 30 s, 85 dB white noise ‘tone’) and unconditioned stimuli (US, a 2 s, 0.5 mA footshock); Phase II) Context tests, 5 min in duration, occurred in the same chambers, while cue tests, consisting of a 3 min baseline (phase III) followed by a 3 min CS presentation (phase IV), took place in a novel context at least 1 h after context tests. The novel context was a ‘disguised’ chamber, which had its color, shape, size, and texture altered using opaque white inserts, as well as different sound (no fans) and odor [cleaned with 70% isopropyl alcohol (Fisher Scientific, Pittsburgh, PA, USA) instead of EtOH, orange extract in box (McCormick Pure Orange Extract, McCormick & Company, Inc., Sparks,MD, USA)], with mice also placed in different chambers (i.e., different locations) under dark conditions (red room lights, no chamber lights).

Weak Trace Fear Conditioning. As we have previously shown minimal genotype differences in trace fear conditioning using 5 CS-US presentations (Clipperton-Allen and Page, 2014), we increased the difficulty of the assay to determine whether it would reveal stronger deficits in the *Pten*^+/-^ mice by only providing a single CS-US presentation (30 s tone, 15 s trace interval, 2 s shock) following a 3 min baseline.

Remote Memory for Trace Fear Conditioning. To determine whether the *Pten*^+/-^ mice had impaired long-term memory, mice were given context and cue tests 30 days after a training program with 3 CS-US pairings (30 s tone, 15 s trace interval, 2 s shock) with 30 s between pairings.

Cued Fear Conditioning Extinction. As this experiment only aimed to condition the mice to the cue, not the context, animals were habituated to the training context in three 4 min sessions the day before training, as previously described (Sillivan et al., 2017). On Day 1, mice were trained as above, except that the CS was a 5 Hz 85 dB tone, coterminating with a 1 s 0.75 mA footshock. In the first cohort, this was presented twice with 2 min between presentations; in the second cohort, only a single pairing was used; as this did not affect freezing or extinction, the two cohorts were combined. After 3 days, on Days 5 and 6, mice received extinction trials consisting of a 2 min baseline followed by 30 CS presentations (in the absence of the US) with 60 s inter-stimulus intervals in a novel context; these presentations were averaged into five 6-presentation bins for each day. Finally, 30 days after training, mice were given a recall test of 5 CS presentations.

##### Three-chamber social approach

This assay was performed under dim white light in the standard manner for our laboratory (Clipperton-Allen et al., 2016; Page et al., 2009a, 2009b) at four ages: juvenile (P23-25), P35, P45, and adult (P64-92). Mice were habituated to the empty apparatus for 5 min on each of three consecutive days. On the third (test) day, two tubes were placed in the two outer chambers, one containing a same-sex stimulus mouse (“mouse + tube chamber”) and one left empty (“empty tube chamber”). Time spent in each chamber was automatically scored by Ethovision XT. The dichotomous social approach variable (the presence or absence of a social preference) was determined by comparing the time spent in the mouse + tube chamber and that spent in the empty tube chamber. Additionally, preference index was calculated to determine the magnitude of the difference in time spent between social and non-social chambers (i.e., the strength of the preference: [(time in mouse + tube chamber) – (time in empty tube chamber)]/[(time in mouse + tube chamber) + (time in empty tube chamber)].

### Quantification and statistical analysis

Pearson’s *r* was used to correlate brain region growth indices for each genotype separately, and one-sample *t*-tests assessed whether brain region size in *Pten*^+/-^ adults differed from that predicted based on *Pten*^+/+^ growth trajectory.

Two-way mixed-model analyses of variance (ANOVAs) were used to analyze AST, ASH/PPI, NOR, puzzle box, OFT, rotarod learning, SSRT, MWM, all three fear conditioning tests, and 3-chamber social approach, with genotype as the between-subjects factor. Within-subjects factors were stimulus (AST) or pre-pulse (PPI) dB, phase (ASH, WFC, RMFC, NOR), condition (puzzle box), thigmotaxis (OFT), day (rotarod learning, SSRT, MWM), extinction bin (FCExt), or chamber type (3-chamber social approach). Two-way between-subjects ANOVAs were used to analyze the effects of age and genotype on the preference index and distance traveled in the experiment investigating the developmental timecourse of 3-chamber social approach. For all two-way ANOVAs, Sidak *post hoc* tests were used when interactions were significant.

Planned comparisons (independent-samples *t*-tests) between genotypes were performed for each stimulus (AST) or pre-pulse (PPI) dB, sigmoid measures (5% startle threshold, midpoint, maximum startle amplitude, sigmoid slope, RMSE; AST), phase (ASH, WFC, RMFC, NOR), DI (NOR), condition (puzzle box), center time (OFT), distance traveled (OFT, 3-chamber social approach), day (rotarod learning), average restricted and free-feeding weight (SSRT), number of success, drop, hit, miss, and “uncounted” reaches (SSRT), first and last cue presentations, extinction bins, extinction score, and recall (FCExt), and preference index (3-chamber social approach). Paired-sample *t*-tests were used as planned comparisons in each sex, genotype, and/or age separately to compare startle amplitude to each stimulus dB to the baseline 70 dB stimulus (AST), novel and familiar objects (NOR), center and thigmotaxis time (OFT), training baseline and context test, and cue baseline and cue test (WFC, RMFC), first and last extinction bin and cue presentation (FCExt), and chambers containing a mouse in a tube and an empty tube (3-chamber social approach). One-way within-subjects ANOVAs were performed separately for each sex and genotype for phase (NOR), day (rotarod learning), training day (SSRT), and extinction bins (cued fear conditioning extinction). For the experiment investigating the developmental timecourse of 3-chamber social approach, one-way between-subjects ANOVAs were used as planned comparisons between ages for the preference index and distance traveled.

One-sample *t*-tests were used in each sex and genotype separately to determine if the mice showed significant normalized startle response amplitude (AST, vs. 0), habituated to startle (ASH, vs. 0) or spent more time in the platform quadrant (MWM, vs. 25% chance), and chi-square tests analyzed the percent of mice completing each trial in the puzzle box assay.

All statistical analyses were performed with PASW 18 (IBM Corporation, Armonk, NY), with significance set at p < 0.05, and p values between 0.05 and 0.10 considered trends. Complete statistical results, including the statistical tests used, are presented in Tables S1, S2, S3, S4, S5, S6, and S7, and thus only select p values are included in the text. The number of animals used (*n*) is indicated on the figures, and all data is reported as mean ± standard error of the mean (SEM).

## Data Availability

All data reported in this paper will be shared by the lead contact upon request. This paper does not report original code. Any additional information required to reanalyze the data reported in this paper is available from the lead contact upon request.

## Supplemental information

Document S1. Figures S1–S8 and Tables S1 –S9

Table S1. Growth index correlations for absolute and relative volume, related to Figure 1 Significant results are in bold, trends are in italics.

Table S3. Statistics for acoustic startle threshold, acoustic startle response, and pre-pulse inhibition tests, related to Figures 2 and 3 Significant results are in bold, trends are in italics.

Table S4. Statistics for novel object recognition, puzzle box, and morris water maze, related to Figures 4 and 5 Significant results are in bold, trends are in italics.

Table S7. Statistics for acoustic startle threshold and three-chamber social approach timecourses, related to Figures 6 and 7 Significant results are in bold, trends are in italics.
