# Supplementary material for: Pten haploinsufficiency causes desynchronized growth of brain areas involved in sensory processing
Source: iScience. 2022 Jan 19;25(2):103796. doi: 10.1016/j.isci.2022.103796 (PMC8844819; doi:10.1016/j.isci.2022.103796)
Supplement: Document S1. Figures S1–S8 and Tables S1 — –S9 [file mmc1.pdf]

iScience, Volume *25*

Supplemental information

Pten haploinsufficiency causes desynchronized growth of brain areas involved in sensory processing

Amy E. Clipperton-Allen, Hannah Swick, Valentina Botero, Massimiliano Aceti, Jacob Ellegood, Jason P. Lerch, and Damon T. Page

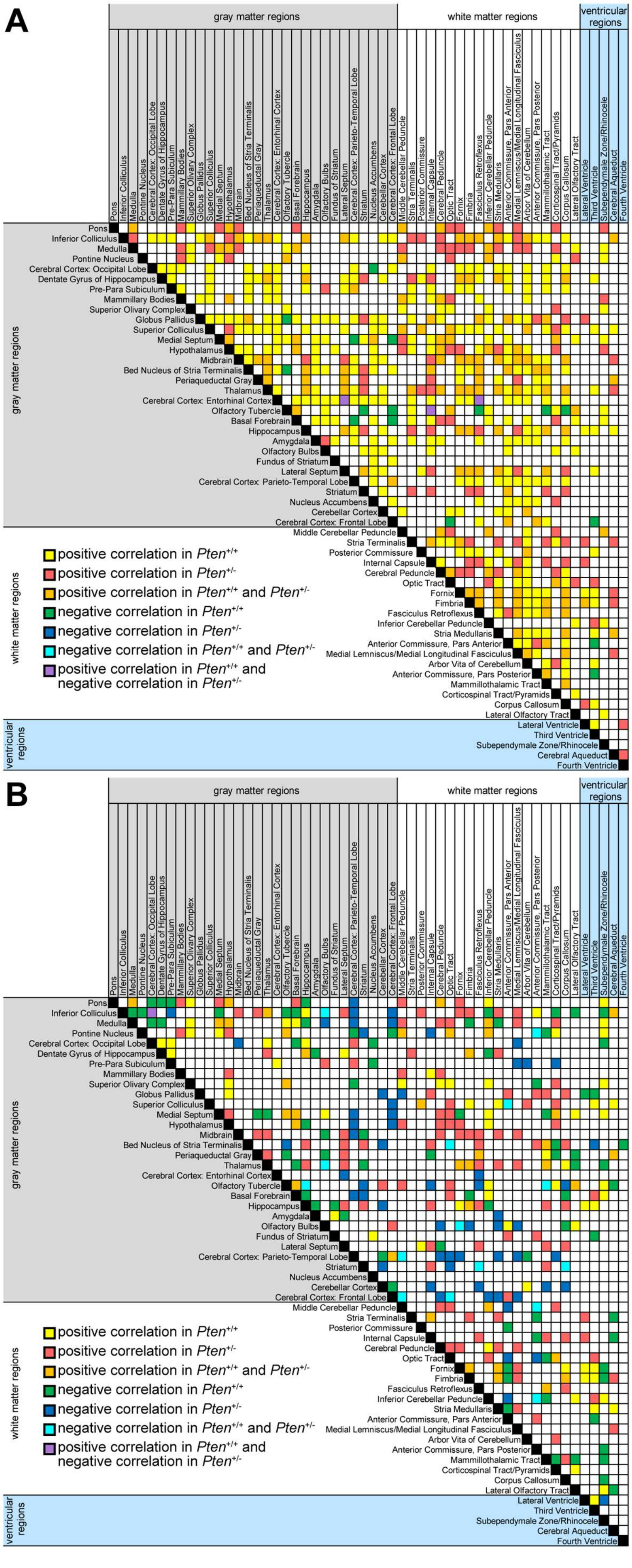

Figure S1. *Pten*$^{+/-}$ mice have abnormal developmental brain region growth trajectories, related to Figure 1. A-B) Correlations between brain region growth indices {[(P60 volume for mouse) – (average P7 volume for genotype)]/(average P7 volume for genotype)} for absolute (**A**) and relative (**B**) volume within genotypes are consistent across genotypes in some regions (orange, positive correlation in both genotypes; cyan, negative correlation in both genotypes) but not others (positive correlations in warm colors: yellow, *Pten*$^{+/+}$ only; red, *Pten*$^{+/-}$ only; negative correlations in cool colors: green, *Pten* $^{+/+}$ only; blue, *Pten*$^{+/-}$ only; purple, correlations that were positive in *Pten*$^{+/+}$ and negative in *Pten*$^{+/-}$ mice). P7, postnatal day 7; P60, postnatal day 60. See also Figure 1, Table S1.

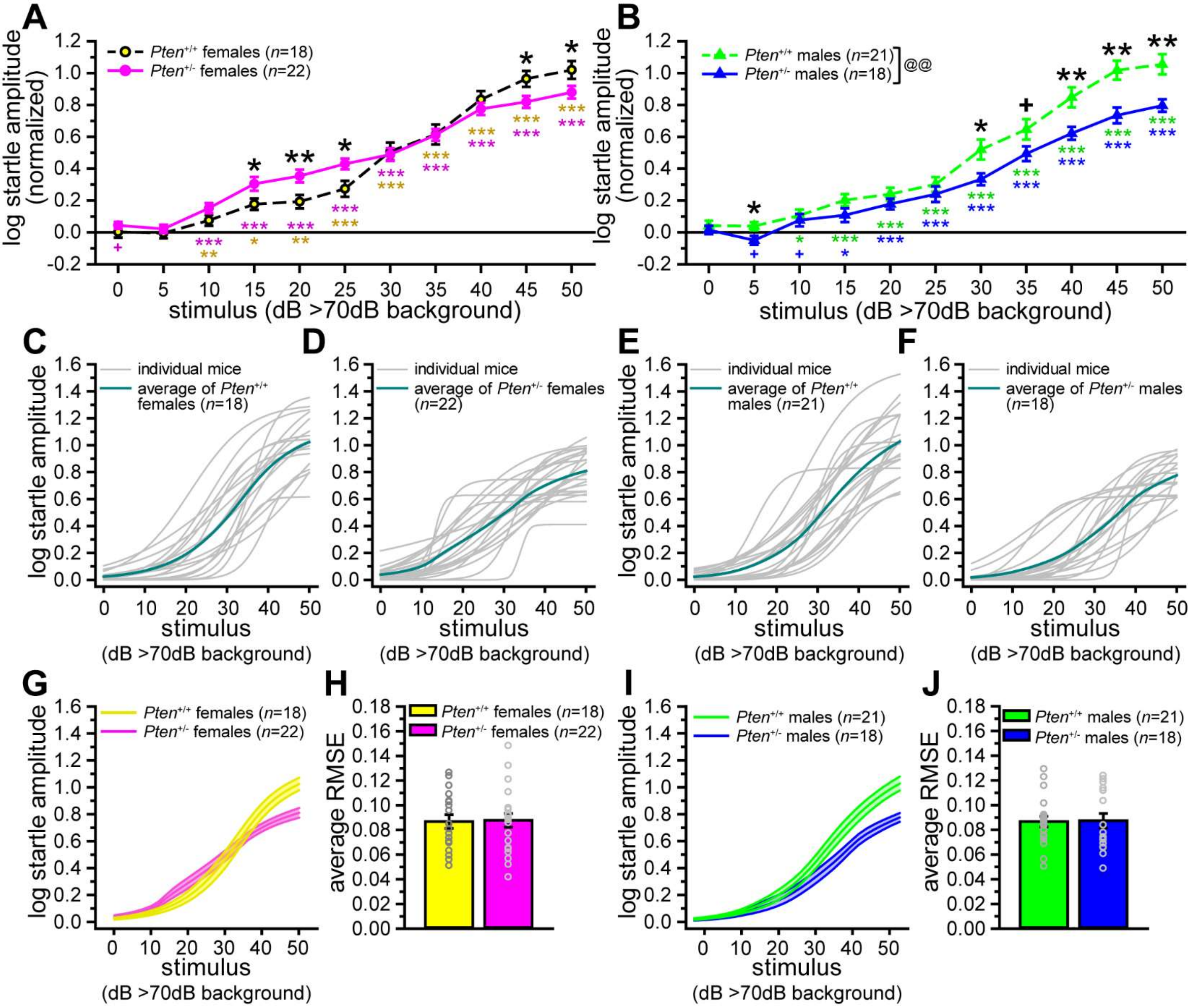

Figure S2. Both sexes of *Pten*$^{+/-}$ mice show hyporeactivity to high-intensity acoustic stimuli, and *Pten*$^{+/-}$ females show hyporeactivity to low-intensity stimuli, related to Figure 2. A) Female *Pten*$^{+/-}$ mice show increased log-normal startle amplitude in response to low-dB stimuli, and decreased log-normal startle amplitude in response to high-dB stimuli. **B**) *Pten*$^{+/-}$ males have decreased log-normal startle amplitude to high-dB stimuli. **C-F**) Individual (gray) and average (teal) sigmoid curves for female *Pten*$^{+/+}$ (**C**) and *Pten*$^{+/-}$ (**D**) mice, and for *Pten*$^{+/+}$ (**E**) and *Pten*$^{+/-}$ (**F**) males. **G**,**I**) Genotype average sigmoid curves for females (**G**) and males (**I**). **H**,**J**) No genotype differences were found for model-fitting error in female (**H**) or male (**J**) mice. RMSE, root mean squared error. Data are represented as mean ± SEM. Black symbols, independent-samples *t*-tests between genotypes. Colored symbols, significant startle (one-sample *t*-tests vs 0). *** p < 0.001, ** p < 0.01, * p < 0.05, + p < 0.10. Main effect of genotype in two-way mixed-model ANOVAs (genotype x stimulus dB): @@ p < 0.01. See also Figure 2, Table S3.

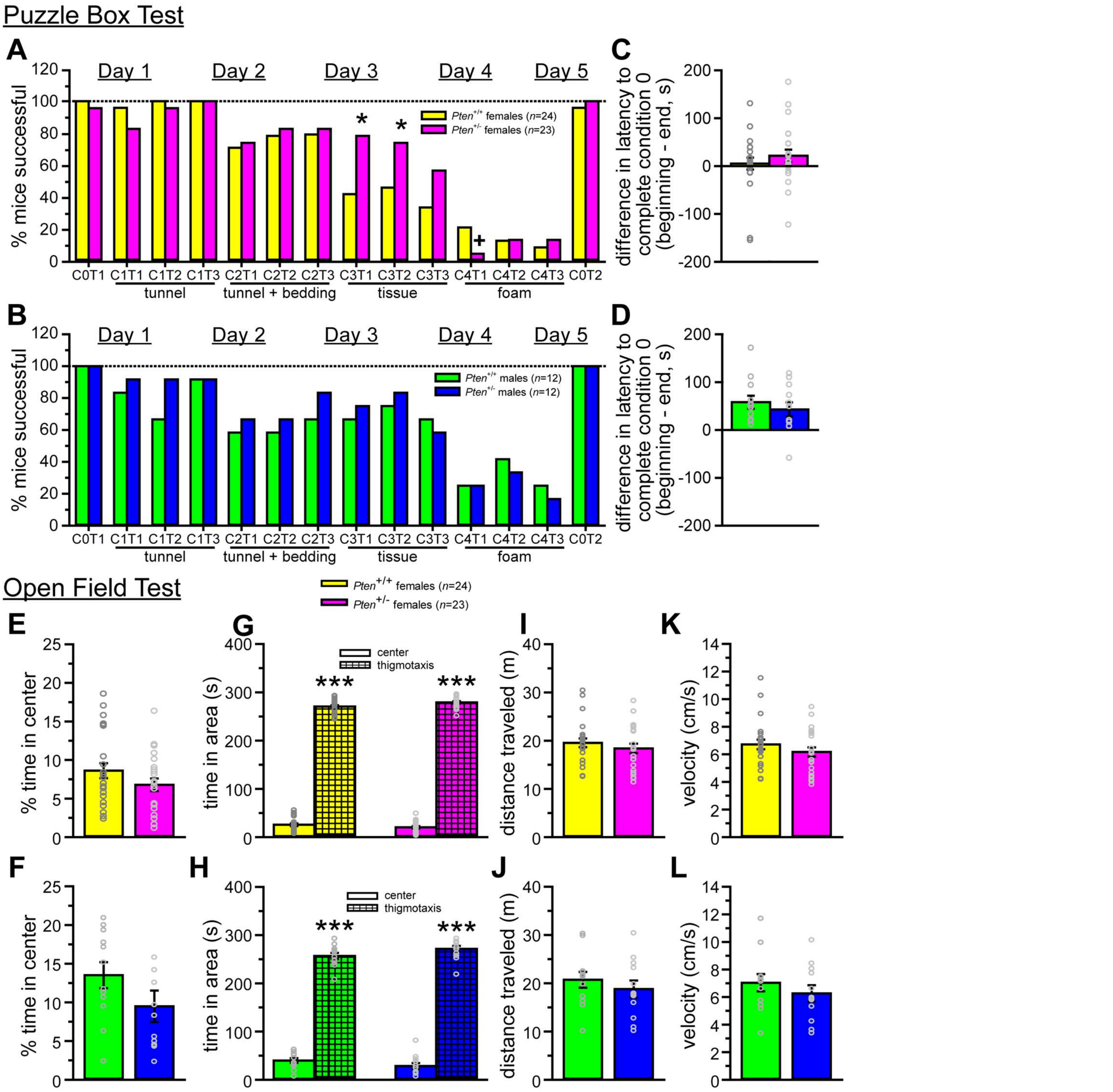

Figure S3. Female *Pten*$^{+/-}$ mice have a higher percentage completion for the tissue condition, but no difference in motivation, locomotion, or anxiety controls, related to Figure 4. A-B) A higher percentage of the female *Pten*$^{+/-}$ than *Pten*$^{+/+}$ mice completed the first two tissue task trials (**A**). There were no genotype differences in the percentage of mice completing any other task in females (**A**) or males (**B**). **C-D**) Neither female (**C**) nor male (**D**) mice of either genotype showed a significant decrease in the latency to complete the no-obstacle condition 0 (C0) from the first (C0T1) to last (C0T2) trial of the puzzle box assay, indicating no reduction in motivation to reach the goal box. C, condition; T, trial. **E-L**) The same mice tested in the puzzle box were given an open field test to ensure there were no locomotor deficits or anxiety phenotypes that could explain or confound the results. There were no differences in center time (**E-F**), distance traveled (**I-J**), or velocity (**K-L**) for either sex, and all groups showed a significant preference for thigmotaxis (**G-H**). Data are represented as mean ± SEM. Black symbols, independent-samples *t*-tests between genotypes (**A-F, I-L)** or thigmotaxis preference (paired-samples *t*-tests between center and thigmotaxis within groups, **G-H**). *** $p < 0.001$, * $p < 0.05$, + $p < 0.10$. See also Figure 4, Table S4.

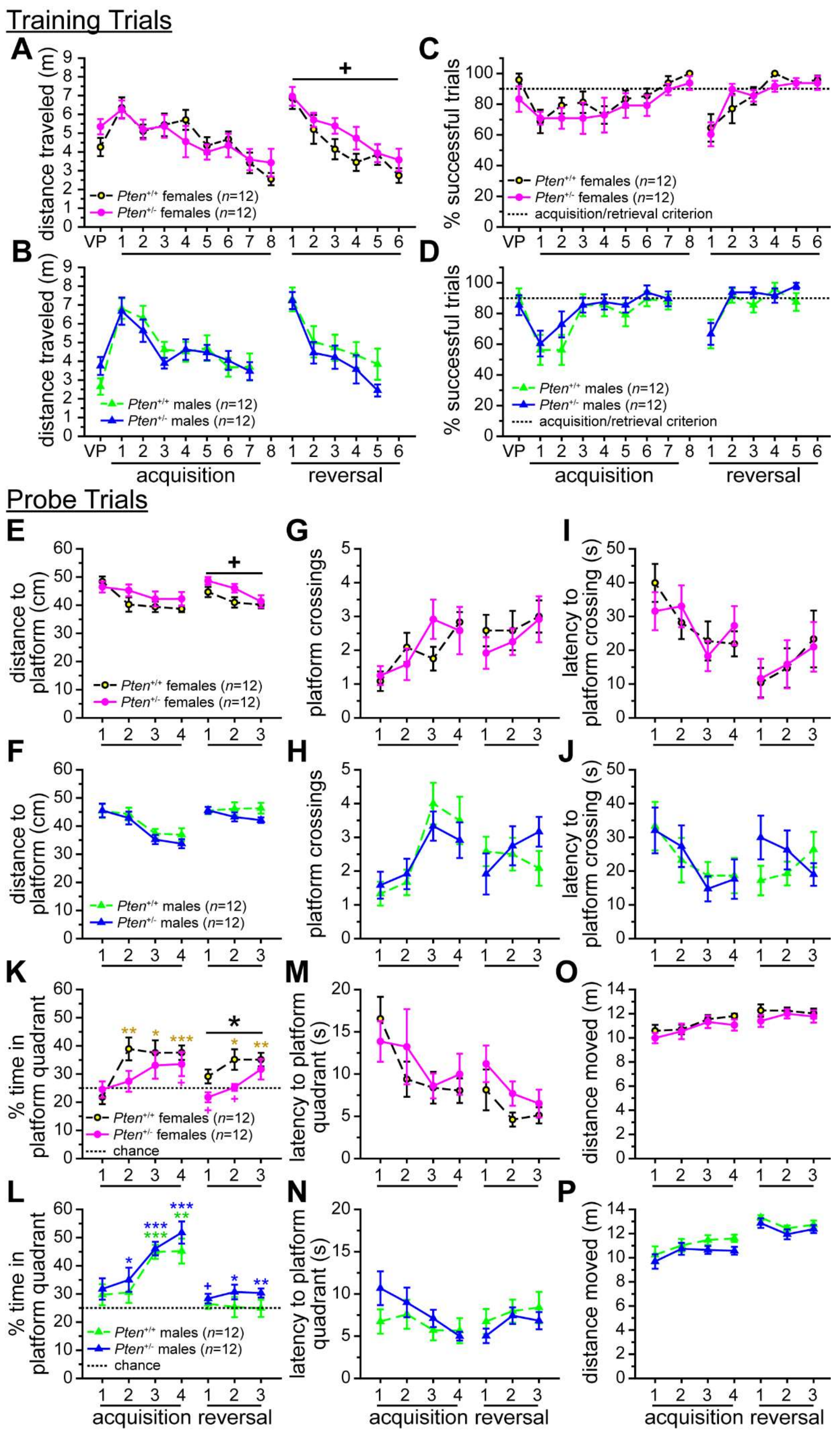

Figure S4. Female *Pten*$^{+/-}$ mice may have subtle spatial memory impairments, but *Pten*$^{+/-}$ mice are largely normal in the Morris water maze, related to Figure 5.
A-D) Female *Pten*$^{+/-}$ mice showed a trend to longer distances traveled than *Pten*$^{+/+}$ females during reversal training (**A**), but no differences in percent successful trials (**C**) for each day of training. *Pten*$^{+/-}$ males had normal distance traveled (**B**) and percent successful trials (**D**) for each day of training. **E-P**) Probe trial results. *Pten*$^{+/-}$ females had a trend to a longer average distance to platform location (**E**) and spent less time in the platform quadrant (**K**) during reversal learning than *Pten*$^{+/+}$ females. Males did not show a difference on either measure (**F,L**). There were no genotype differences in either sex for platform crossings (**G,H**), latency to cross platform location (**I,J**), or latency to the platform quadrant (**M,N**). No female genotype differences were found for total distance moved (**O**) during probe trials, although male *Pten*$^{+/-}$ mice traveled a shorter distance than *Pten*$^{+/+}$ males in the final probe trial of acquisition (**P**). VP, visual platform test. Data are represented as mean ± SEM. Black symbols, main effect of genotype in two-way mixed-model ANOVAs (genotype x day, **A-N**) or independent-samples *t*-tests between genotypes (**O-P**). Colored symbols, difference from chance (one-sample *t*-tests vs. 25%). Dashed line, acquisition or retrieval criterion (**C-D**), chance (**K-L**). *** $p < 0.001$, ** $p < 0.01$, * $p < 0.05$, + $p < 0.10$. See also Figure 5, Table S4.

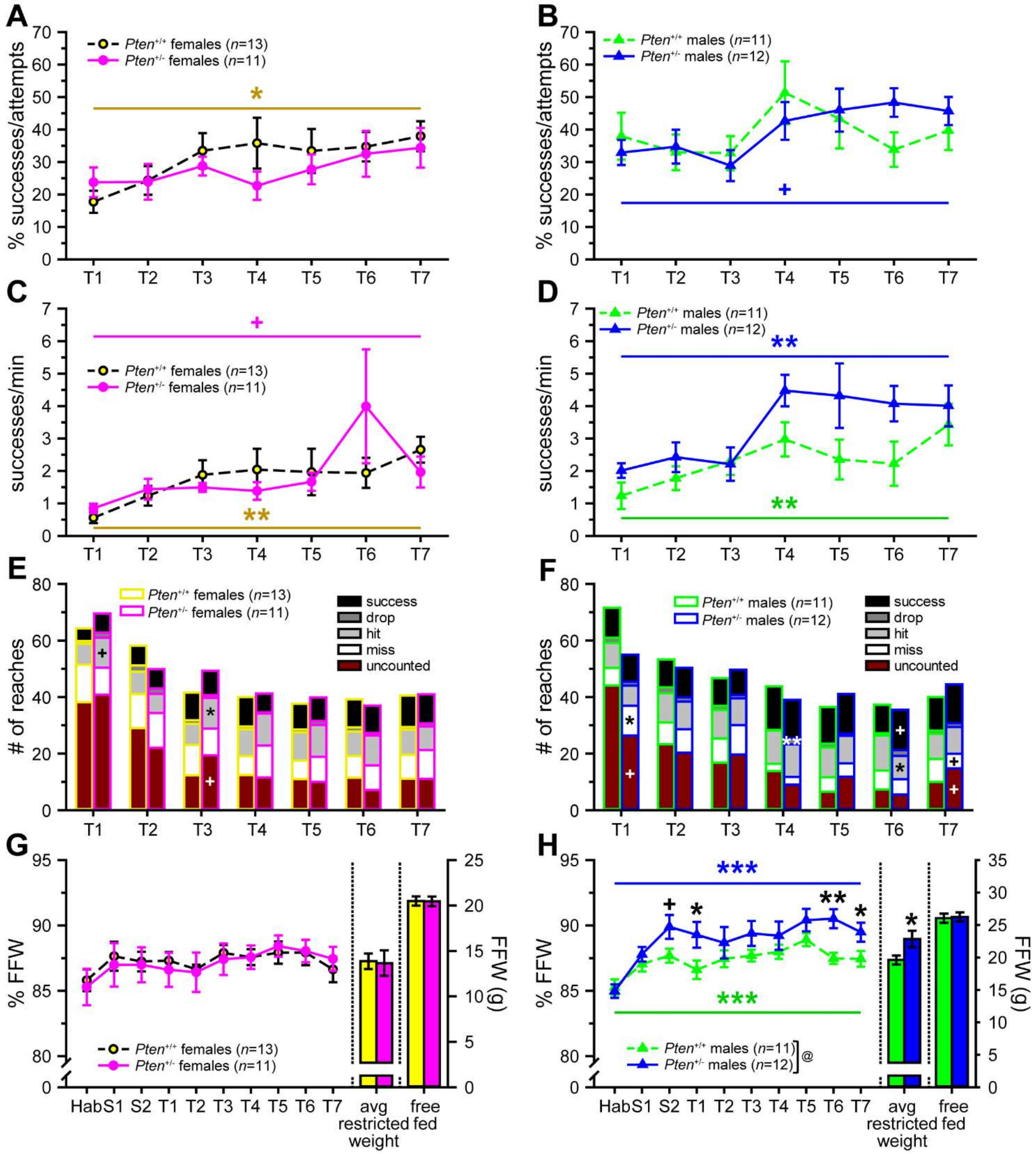

Figure S5. *Pten*$^{+/-}$ mice show few differences in the single-seed reaching task, related to Figure 5. A-B) Female *Pten*$^{+/+}$ but not *Pten*$^{+/-}$ (**A**) and male *Pten*$^{+/-}$ but not *Pten*$^{+/+}$ mice (**B**) showed improvement over training for the percent of attempts that were successes. **C-D**) All female (**C**) and male (**D**) mice improved the number of successful attempts per minute over training. **E-F**) Overview of reach types across training. Female *Pten*$^{+/-}$ mice made more hits during training day 1 (T1) and T3, and more uncounted reaches during T3, than *Pten*$^{+/+}$ mice (**E**). Male *Pten*$^{+/-}$ mice made more misses during T1, drops during T4, successes during T6, and uncounted reaches during T7, but fewer uncounted reaches during T1, hits during T6, and misses during T7, than *Pten*$^{+/+}$ males (**F**). **G-H**) Female *Pten*$^{+/-}$ mice showed normal weight before and during food restriction (**G**). Male *Pten*$^{+/-}$ mice showed no weight difference before food restriction, but significantly higher restricted weight in comparison to *Pten*$^{+/+}$ males, and both genotypes showed an increase in restricted weight across testing (**H**). Hab, habituation day; S1, shaping day 1; S2, shaping day 2. Data are represented as mean ± SEM. Black or white symbols, independent-samples *t*-tests between genotypes. Colored symbols, change over time (one-way within-subjects ANOVAs). *** $p < 0.001$, ** $p < 0.01$, * $p < 0.05$, + $p < 0.10$. Main effect of genotype in two-way mixed-model ANOVAs (genotype x day): @ $p < 0.05$. See also Figure 5, Table S5.

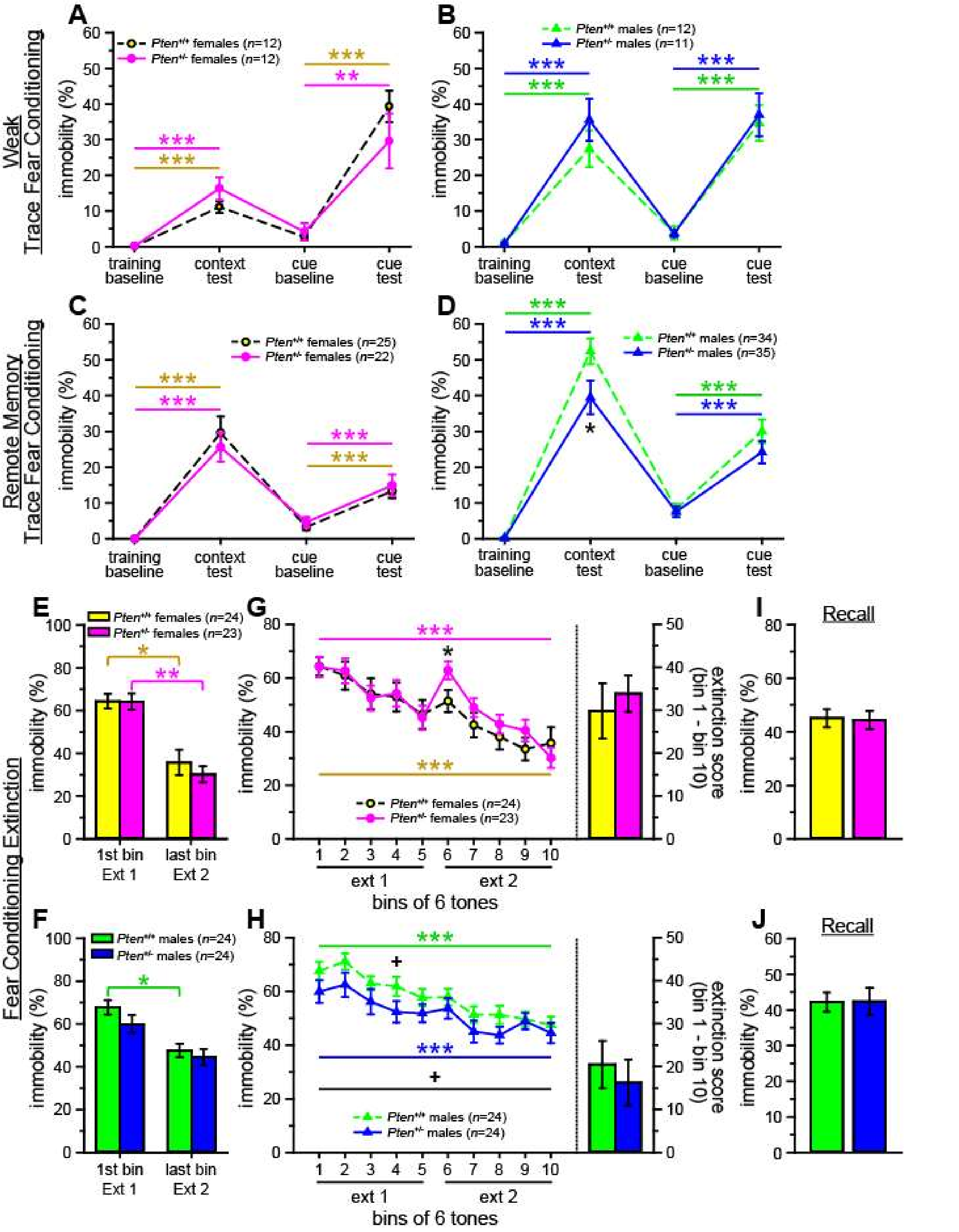

Figure S6. *Pten*$^{+/-}$ mice show largely normal fear conditioning and extinction learning, related to Figure 5. A-B) No genotype differences were found for female (**A**) or male (**B**) mice during any phase of weak (single CS-US pairing) trace fear conditioning. **C-D**) Male *Pten*$^{+/-}$ mice froze less than *Pten*$^{+/+}$ during the context test (**D**), but no other genotype differences were found for females (**C**) or males (**D**) when tested 30 days after training. **E-J**) Most groups showed extinction, with reductions in freezing from first to last CS presentation except in *Pten*$^{+/-}$ males (**E,F**) and in all groups across extinction bins of 6 tones (**G,H**). Female *Pten*$^{+/-}$ mice froze more during the first bin of extinction trial 2 (**G**), but showed no other genotype differences. *Pten*$^{+/-}$ males froze less during extinction trial 1 bin 4 (**H**). No genotype differences were found for extinction score (**G,H**) or recall (**I,J**). CS+, cue presentation; Ext, extinction day; bin, average of 6 extinction trials. Data are represented as mean ± SEM. Black symbols, independent-samples *t*-tests between genotypes. Colored symbols, baseline vs. test paired-samples *t*-tests (**A-D**) or change over time (one-way within-subjects ANOVAs, **E-J**). *** $p < 0.001$, ** $p < 0.01$, * $p < 0.05$, + $p < 0.1$. See also Figure 5, Table S6.

A

	Tested Behavior		Brain Region	MRI Region	*Pten*$^{+/-}$ Effect (Rel)[1]	Dev. From Predicted (Rel)[2a]
Behaviors with Significant Phenotypes	Acoustic Startle Threshold[2b]	♀ ↑/↓<br>♂ ↓	Inferior Colliculus, Medulla		↑	↑
			Caudal Nucleus of Pontine Reticular Formation (PnC)	Pons<br>Pontine Nucleus	↑<br>—	↑<br>—
			Superior Olivary Complex		—	—
	Acoustic Startle Response[2c]	♀ ↓<br>♂ ↓	Inferior Colliculus, Medulla		↑	↑
			Caudal Nucleus of Pontine Reticular Formation (PnC)	Pons<br>Pontine Nucleus	↑<br>—	↑<br>—
	Pre-pulse Inhibition[2d,3]	♀ ↓<br>♂ ↓	Inferior Colliculus, Superior Colliculus, Medulla		↑	↑
			Caudal Nucleus of Pontine Reticular Formation (PnC)<br>Laterodorsal Tegmental Nucleus (LDTg)	Pons<br>Pontine Nucleus	↑<br>—	↑<br>—
			Pedunculopontine Tegmental Nucleus (PPTg)<br>Substantia Nigra, Pars Reticulata (SNR)	Midbrain	—	—
	Marble Burying[4]	♀ –<br>♂ ↑	Globus Pallidus		↑	↑
			Striatum		—	↓
			Hippocampus, Thalamus		—	—
			Substantia Nigra, Pars Reticulata (SNR)	Midbrain	—	—
	Social Approach[2e,3]	♀ ↓<br>♂ –	Hypothalamus		—	↑
			Anterior Cingulate, Orbitofrontal, Prefrontal Cortices	Frontal Lobe	↓	↓
			Olfactory Bulbs, Nucleus Accumbens		↓	↓
			Amygdala*, Striatum		—	↓
			Hippocampus		—	—
	Social Recognition[4]	♀ –<br>♂ ↓	Hypothalamus, Medial Septum		—	↑
			Anterior Cingulate, Orbitofrontal, Prefrontal Cortices	Frontal Lobe	↓	↓
			Piriform Cortex	Parieto-Temporal Lobe	↓	↓
			Amygdala*, Lateral Septum*		—	↓
			Hippocampus		—	—
	Aggression[5]	♀ n/a<br>♂ ↓	Hypothalamus, Medial Septum		—	↑
			Medial Preoptic Area	Hypothalamus	—	↑
			Anterior Cingulate Cortex	Frontal Lobe	↓	↓
			Insular Cortex	Parieto-Temporal Lobe	↓	↓
			Amygdala*, Striatum, Lateral Septum*		—	↓
			Bed Nucleus of the Stria Terminalis, Hippocampus, Periaqueductal Grey, Thalamus		—	—
			Dorsal Raphe	Midbrain	—	—
	Puzzle Box[2f]	♀ ↑<br>♂ –	Hypothalamus		—	↑
			Anterior Cingulate, Prefrontal Cortices	Frontal Lobe	↓	↓
			Cerebellum	Cerebellar Cortex	—	↓
			Hippocampus, Thalamus		—	—
	NOR[2g]	♀ ↓<br>♂ –	Anterior Cingulate, Prefrontal Cortices	Frontal Lobe	↓	↓
			Perirhinal Cortex	Parieto-Temporal Lobe	↓	↓
			Entorhinal Cortex, Hippocampus		—	—
	Anxiety[4]	♀ –<br>♂ ↓	Raphe nuclei	Midbrain<br>Medulla<br>Pons<br>Pontine Nucleus	—<br>—<br>↑<br>—	—<br>↑<br>↑<br>—
			Medial Septum		—	↑
			Prefrontal Cortex	Frontal Lobe	↓	↓
			Amygdala*, Lateral Septum*		—	↓
			Hippocampus		—	—
	Depression[4]	♀ –<br>♂ ↑	Raphe nuclei	Midbrain<br>Medulla<br>Pons<br>Pontine Nucleus	—<br>—<br>↑<br>—	—<br>↑<br>↑<br>—
			Hypothalamus, Medial Septum		—	↑
			Prefrontal, Anterior Cingulate Cortices	Frontal Lobe	↓	↓
			Nucleus Accumbens		↓	↓
			Amygdala*, Striatum, Lateral Septum*		—	↓
			Hippocampus, Thalamus		—	—
	Remote Memory FC[2h]	♀ –<br>♂ ↑/–	Hypothalamus		—	↑
			Prefrontal, Anterior Cingulate Cortices	Frontal Lobe	↓	↓
			Amygdala*, Striatum, Lateral Septum*		—	↓
			Entorhinal Cortex, Hippocampus, Thalamus		—	—
Behaviors without Signifciant Phenotypes	Rotarod Learning[2i]	♀ –<br>♂ –	Motor, Prefrontal, Frontal Cortices	Frontal Lobe	↓	↓
			Amygdala*, Striatum		—	↓
			Cerebellum	Cerebellar Cortex	—	↓
			Hippocampus, Thalamus		—	—
	SSRT[2j]	♀ –<br>♂ –	Medulla		↑	↑
			Motor Cortex	Frontal Lobe	↓	↓
			Cerebellum	Cerebellar Cortex	—	↓
			Striatum		—	↓
			Thalamus		—	—
	MWM[2k]	♀ –<br>♂ –	Prefrontal Cortex	Frontal Lobe	↓	↓
			Perirhinal Cortex	Parieto-Temporal Lobe	↓	↓
			Cerebellum	Cerebellar Cortex	—	↓
			Striatum		—	↓
			Entorhinal Cortex, Hippocampus		—	—
	Spont. Alternation[6]	♀ –<br>♂ –	Medial Raphe	Pons	↑	↑
			Medial Septum		—	↑
			Prefrontal Cortex	Frontal Lobe	↓	↓
			Striatum, Lateral Septum*		—	↓
			Cerebellum	Cerebellar Cortex	—	↓
			Hippocampus, Thalamus		—	—
	Trace FC[2l]	♀ –<br>♂ –	Anterior Cingulate Cortex	Frontal Lobe	↓	↓
			Amygdala*		—	↓
			Entorhinal Cortex, Hippocampus, Thalamus		—	—
	FC Extinction[2m]	♀ –<br>♂ –	Prefrontal, Anterior Cingulate Cortices	Frontal Lobe	↓	↓
			Amygdala*		—	↓
			Thalamus		—	—

brain region(s) increased (red) · brain region(s) decreased (blue) · brain regions unchanged (white)

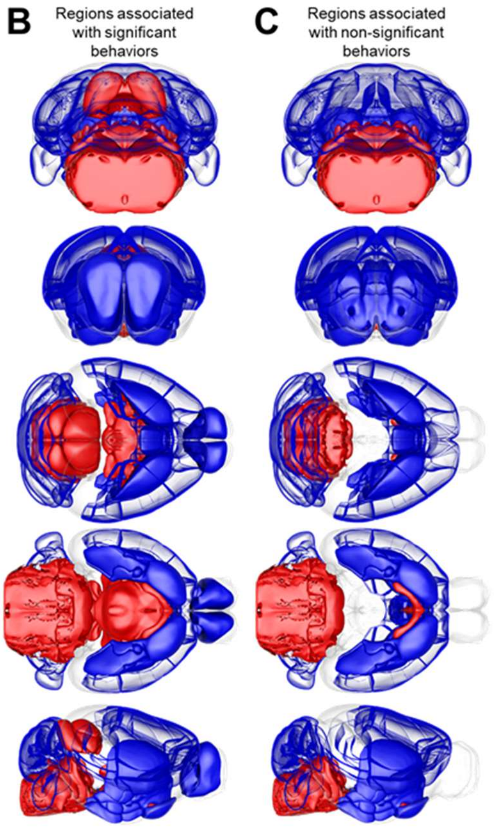

Figure S7. Brain regions that are relatively enlarged or have increased growth trajectories predict behavioral phenotypes, related to Figures 1-5, 7. A) Table of behaviors showing significant phenotypes in at least one sex (upper, black) or no phenotype (lower, white) and associated brain regions. **B-C**) Visual representation of brain areas associated with behaviors showing a significant phenotype (**B**) or no phenotype (**C**).*, statistical trend. Red, increased relative volume (Clipperton-Allen et al., 2019) and/or positive deviation from predicted relative growth trajectory **(Fig. 1E)**; blue, decreased relative volume (Clipperton-Allen et al., 2019) and/or negative deviation from predicted relative growth trajectory (**Fig. 1E**); white, no significant change in relative volume (Clipperton-Allen et al., 2019) and no significant deviation from predicted growth trajectory (**Fig. 1E**). *Pten*$^{+/-}$Effect (Rel.), direction for significant relative volume differences between adult *Pten*$^{+/+}$ and *Pten*$^{+/-}$ mice in Clipperton-Allen et al., 2019; Dev. From Predicted (Rel.), direction for significant deviations from relative *Pten*$^{+/+}$ growth trajectory (**Fig. 1C,E**); NOR, novel object recognition; SSRT, single-seed reaching task; MWM, Morris water maze; Spont. Alternation, spontaneous alternation; FC, fear conditioning. Brain scaling and behavior results based on data from: [1] Clipperton-Allen et al., 2019; [2] current paper ([a] Fig. 1D,F; [b] Fig. 2B-C; [c] Fig. 3B-C; [d] Fig. 3E-F; [e] Fig. 7G-H; [f] Fig. 4G-H, Fig. S1A-B; [g] Fig. 4B-C; [h] Fig. 5I-J, Fig. S4C-D; [i] Fig. 5C-D; [j] Fig. 5E-F, Fig. S3A-F; [k] Fig. 5A-B, Fig. S2; [l] Fig. 5G-H, Fig. S4A-B; [m] Fig. 5K-L, Fig. S4E-J); [3] Page et al., 2009a; [4] Clipperton-Allen and Page, 2014; [5] Clipperton-Allen and Page, 2015; [6] Huang et al., 2016.

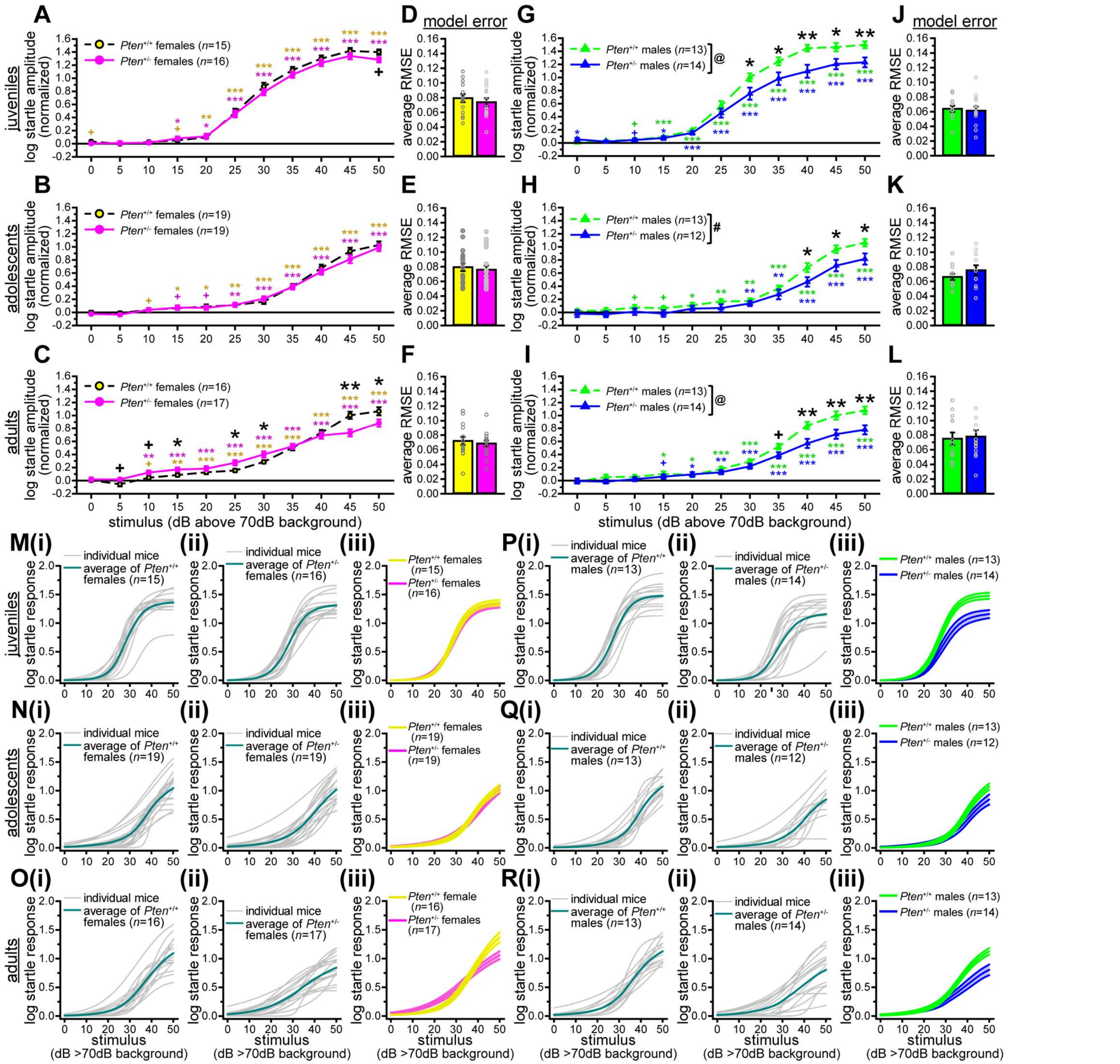

Fig. S8. Male and female juvenile *Pten*$^{+/-}$ mice show different aspects of adult acoustic startle threshold phenotypes, related to Figure 6. A-C) Adult female *Pten*$^{+/-}$ mice show increased log-normal startle amplitude in response to low-dB stimuli, and decreased log-normal startle amplitude in response to high-dB stimuli (**C**), but minimal differences were found in juvenile (**A**) or adolescent (**B**) females. **D-F**) No genotype differences were found in model fitting error for female juvenile (**D**), adolescent (**E**), or adult (**F**) mice. **G-I**) Juvenile (**G**), adolescent (**H**), and adult (**I**) *Pten*$^{+/-}$ males have decreased log-normal startle amplitude to high-dB stimuli. **J-L**) Model fitting error did not differ between genotypes for male juveniles (**J**), adolescents (**K**), or adults (**L**). **M-O**) Individual (gray) and average (teal) sigmoid curves for female juveniles [*Pten*$^{+/+}$, **M(i)**; *Pten*$^{+/-}$, **M(ii)**], adolescents [*Pten*$^{+/+}$, **N(i)**; *Pten*$^{+/-}$, **N(ii)**], and adults [*Pten*$^{+/+}$, **O(i)**; *Pten*$^{+/-}$, **O(ii)**], with genotype averages [juveniles, **M(iii)**; adolescents, **N(iii)**; adults, **O(iii)**]. **P-R**) Individual (gray) and average (teal) sigmoid curves for male juveniles [*Pten*$^{+/+}$, **P(i)**; *Pten*$^{+/-}$, **P(ii)**], adolescents [*Pten*$^{+/+}$, **Q(i)**; *Pten*$^{+/-}$, **Q(ii)**], and adults [*Pten*$^{+/+}$, **R(i)**; *Pten*$^{+/-}$, **R(ii)**], with genotype averages [juveniles, **P(iii)**; adolescents, **Q(iii)**; adults, **R(iii)**]. RMSE, root mean squared error. Data are represented as mean ± SEM. Black symbols, independent-samples *t*-tests between genotypes. Colored symbols, significant startle (one-sample *t*-tests vs. 0). *** $p < 0.001$, ** $p < 0.01$, * $p < 0.05$, + $p < 0.1$. See also Figure 6, Table S7.

Table S2. Statistics for *Pten*$^{+/-}$ deviation from *Pten*$^{+/+}$ growth trajectory, related to Figure 1.

Analysis	Brain Region	Absolute Volume	Relative Volume
One-sample *t*-test: % deviation from *Pten*$^{+/+}$ growth trajectory	Pons	**$t(8)=12.8, p<0.001$**	**$t(8)=9.10, p<0.001$**
	Inferior Colliculus	**$t(8)=9.32, p<0.001$**	**$t(8)=5.89, p<0.001$**
	Medulla	**$t(8)=9.24, p<0.001$**	**$t(8)=6.24, p<0.001$**
	Pontine Nucleus	**$t(8)=3.83, p=0.005$**	$t(8)=1.77, p=0.115$
	Cerebral Cortex: Occipital Lobe	**$t(8)=8.99, p<0.001$**	**$t(8)=3.54, p=0.008$**
	Dentate Gyrus Of Hippocampus	**$t(8)=8.86, p<0.001$**	**$t(8)=4.56, p=0.002$**
	Mammillary Bodies	**$t(8)=5.27, p=0.001$**	**$t(8)=2.36, p=0.046$**
	Pre-Para Subiculum	**$t(8)=19.33, p<0.001$**	**$t(8)=8.13, p<0.001$**
	Superior Olivary Complex	**$t(8)=3.97, p=0.004$**	$t(8)=1.51, p=0.169$
	Globus Pallidus	**$t(8)=9.28, p<0.001$**	**$t(8)=3.68, p=0.006$**
	Superior Colliculus	**$t(8)=7.16, p<0.001$**	**$t(8)=3.30, p=0.011$**
	Medial Septum	**$t(8)=7.17, p<0.001$**	**$t(8)=2.60, p=0.031$**
	Hypothalamus	**$t(8)=8.66, p<0.001$**	**$t(8)=2.63, p=0.030$**
	Midbrain	**$t(8)=5.79, p<0.001$**	$t(8)=1.82, p=0.107$
	Bed Nucleus Of Stria Terminalis	**$t(8)=4.55, p=0.002$**	$t(8)=0.94, p=0.374$
	Periaqueductal Grey	**$t(8)=3.91, p=0.004$**	$t(8)=0.46, p=0.660$
	Thalamus	**$t(8)=5.78, p<0.001$**	$t(8)=0.83, p=0.431$
	Olfactory Tubercle	**$t(8)=3.68, p=0.006$**	$t(8)=0.28, p=0.783$
	Cerebral Cortex: Entorhinal Cortex	**$t(8)=8.50, p<0.001$**	$t(8)=0.65, p=0.534$
	Basal Forebrain	**$t(8)=6.36, p<0.001$**	$t(8)=0.20, p=0.849$
	Hippocampus	**$t(8)=6.76, p<0.001$**	$t(8)=0.12, p=0.911$
	Olfactory Bulbs	**$t(8)=13.96, p<0.001$**	**$t(8)=3.83, p=0.005$**
	Amygdala	**$t(8)=5.79, p<0.001$**	*$t(8)=1.98, p=0.082$*
	Fundus Of Striatum	**$t(8)=4.49, p=0.002$**	$t(8)=1.47, p=0.179$
	Cerebral Cortex: Parieto-Temporal Lobe	**$t(8)=4.87, p=0.001$**	**$t(8)=2.90, p=0.020$**
	Lateral Septum	**$t(8)=3.29, p=0.011$**	*$t(8)=2.16, p=0.063$*
	Striatum	**$t(8)=5.39, p=0.001$**	**$t(8)=4.73, p=0.001$**
	Nucleus Accumbens	**$t(8)=5.48, p=0.001$**	**$t(8)=3.71, p=0.006$**
	Cerebellar Cortex	$t(8)=1.51, p=0.170$	**$t(8)=2.90, p=0.020$**
	Cerebral Cortex: Frontal Lobe	$t(8)=1.55, p=0.160$	**$t(8)=7.36, p<0.001$**
	Middle Cerebellar Peduncle	**$t(8)=9.86, p<0.001$**	**$t(8)=6.75, p<0.001$**
	Stria Terminalis	**$t(8)=16.55, p<0.001$**	**$t(8)=13.37, p<0.001$**
	Posterior Commissure	**$t(8)=10.76, p<0.001$**	**$t(8)=7.82, p<0.001$**
	Internal Capsule	**$t(8)=15.00, p<0.001$**	**$t(8)=9.84, p<0.001$**
	Cerebral Peduncle	**$t(8)=8.89, p<0.001$**	**$t(8)=5.77, p<0.001$**
	Optic Tract	**$t(8)=7.05, p<0.001$**	**$t(8)=3.94, p=0.004$**
	Fornix	**$t(8)=9.91, p<0.001$**	**$t(8)=5.86, p<0.001$**
	Fimbria	**$t(8)=6.63, p<0.001$**	**$t(8)=3.72, p=0.006$**
	Fasciculus Retroflexus	**$t(8)=6.08, p<0.001$**	**$t(8)=2.99, p=0.017$**
	Inferior Cerebellar Peduncle	**$t(8)=7.15, p<0.001$**	**$t(8)=3.46, p=0.009$**
	Stria Medullaris	**$t(8)=7.74, p<0.001$**	**$t(8)=3.76, p=0.006$**
	Anterior Commissure, Pars Anterior	**$t(8)=14.53, p<0.001$**	**$t(8)=5.17, p=0.001$**
	Medial Lemniscus/Medial Longitudinal Fasciculus	**$t(8)=5.32, p=0.001$**	$t(8)=1.72, p=0.123$
	Arbor Vita of Cerebellum	**$t(8)=7.69, p<0.001$**	**$t(8)=2.60, p=0.031$**
	Anterior Commissure, Pars Posterior	**$t(8)=5.54, p=0.001$**	$t(8)=0.50, p=0.632$
	Mammillothalamic Tract	**$t(8)=5.17, p=0.001$**	$t(8)=0.53, p=0.612$
	Corticospinal Tract/Pyramids	**$t(8)=3.86, p=0.005$**	$t(8)=0.26, p=0.801$
	Corpus Callosum	**$t(8)=6.58, p<0.001$**	$t(8)=0.61, p=0.561$
	Lateral Olfactory Tract	**$t(8)=10.88, p<0.001$**	$t(8)=0.81, p=0.443$
	Lateral Ventricle	**$t(8)=8.90, p<0.001$**	**$t(8)=6.40, p<0.001$**
	Third Ventricle	**$t(8)=7.67, p<0.001$**	**$t(8)=3.87, p=0.005$**
	Subependymale Zone/Rhinocele	**$t(8)=14.00, p<0.001$**	**$t(8)=4.37, p=0.002$**
	Cerebral Aqueduct	**$t(8)=3.21, p=0.013$**	$t(8)=1.04, p=0.327$
	Fourth Ventricle	**$t(8)=3.93, p=0.004$**	$t(8)=0.99, p=0.350$

Significant results are in bold, trends are in italics.

Table S5. Statistics for Rotarod Learning and Single-Seed Reaching Task, related to Figure 5.

Rotarod Learning			
Analysis	Effect	Statistics in Females	Statistics in Males
4 (day) x 2 (genotype) ANOVA	genotype	$F(1,23)=0.23$, $p=0.640$	$F(1,22)=0.19$, $p=0.668$
	day	**$F(3,69)=22.22$, $p<0.001$**	**$F(3,66)=16.40$, $p<0.001$**
	genotype x day	$F(3,69)=1.25$, $p=0.299$	$F(3,66)=0.09$, $p=0.963$
One-way (day) ANOVA	$Pten^{+/+}$	**$F(3,33)=8.65$, $p<0.001$**	**$F(3,33)=18.10$, $p<0.001$**
	$Pten^{+/-}$	**$F(3,36)=14.23$, $p<0.001$**	**$F(3,33)=5.15$, $p=0.005$**
Genotype *t*-test	Day 1	**$t(23)=2.11$, $p=0.046$**	$t(22)=0.40$, $p=0.695$
	Day 2	$t(23)=0.09$, $p=0.932$	$t(22)=0.35$, $p=0.729$
	Day 3	$t(23)=0.16$, $p=0.871$	$t(22)=0.57$, $p=0.573$
	Day 10	$t(23)=0.33$, $p=0.743$	$t(22)=0.03$, $p=0.974$

Single-Seed Reaching Task			
Analysis	Effect	Statistics in Females	Statistics in Males
7 (training day) x 2 (genotype) ANOVA for total reaches	genotype	$F(1,12)=0.09$, $p=0.771$	$F(1,8)=1.67$, $p=0.233$
	day	**$F(6,72)=5.62$, $p<0.001$**	**$F(6,48)=12.51$, $p<0.001$**
	genotype x day	$F(6,72)=0.25$, $p=0.959$	**$F(6,48)=3.87$, $p=0.003$**
	post hoc (Sidak): $Pten^{+/+}$ vs $Pten^{+/-}$	n/a	**Training Day 1: $p=0.005$**
		n/a	Training Day 2: $p=0.844$
		n/a	Training Day 3: $p>0.999$
		n/a	Training Day 4: $p=0.405$
		n/a	Training Day 5: $p=0.136$
		n/a	Training Day 6: $p=0.651$
		n/a	Training Day 7: $p=0.168$
7 (training day) x 2 (genotype) ANOVA for % successful attempts	genotype	$F(1,11)=0.06$, $p=0.810$	$F(1,8)<0.01$, $p=0.972$
	day	**$F(6,66)=3.04$, $p=0.011$**	**$F(6,48)=2.40$, $p=0.042$**
	genotype x day	$F(6,66)=1.67$, $p=0.141$	$F(6,48)=1.63$, $p=0.160$
7 (training day) x 2 (genotype) ANOVA for successes/min	genotype	$F(1,11)=0.01$, $p=0.909$	$F(1,8)=3.08$, $p=0.117$
	day	**$F(6,66)=3.67$, $p=0.003$**	**$F(6,48)=8.08$, $p<0.001$**
	genotype x day	$F(6,66)=1.26$, $p=0.290$	$F(6,48)=0.46$, $p=0.834$
10 (habituation, shaping, training) x 2 (genotype) ANOVA for % FFW	genotype	$F(1,22)=0.02$, $p=0.899$	**$F(1,21)=4.68$, $p=0.042$**
	day	**$F(9,198)=2.58$, $p=0.008$**	**$F(9,189)=9.26$, $p<0.001$**
	genotype x day	$F(9,198)=0.28$, $p=0.980$	$F(9,189)=1.43$, $p=0.177$
One-way (training day) ANOVA for total reaches	$Pten^{+/+}$	**$F(6,36)=3.25$, $p=0.012$**	**$F(6,24)=11.41$, $p<0.001$**
	$Pten^{+/-}$	**$F(6,36)=2.72$, $p=0.028$**	**$F(6,24)=3.14$, $p=0.020$**
One-way (training day) ANOVA for % successful attempts	$Pten^{+/+}$	**$F(6,30)=3.38$, $p=0.012$**	$F(6,24)=1.90$, $p=0.122$
	$Pten^{+/-}$	$F(6,36)=0.92$, $p=0.492$	*$F(6,24)=2.16$, $p=0.084$*
One-way (training day) ANOVA for successes/min	$Pten^{+/+}$	**$F(6,30)=5.15$, $p=0.001$**	**$F(6,24)=4.00$, $p=0.006$**
	$Pten^{+/-}$	*$F(6,36)=2.22$, $p=0.063$*	**$F(6,24)=4.48$, $p=0.004$**
One-way (training day) ANOVA for % FFW	$Pten^{+/+}$	$F(9,108)=1.55$, $p=0.141$	**$F(9,90)=3.93$, $p<0.001$**
	$Pten^{+/-}$	$F(9,90)=1.28$, $p=0.258$	**$F(9,99)=6.39$, $p<0.001$**
Genotype *t*-test	successes: T1	$t(15)=1.33$, $p=0.204$	$t(13)=0.38$, $p=0.712$
	successes: T2	$t(20)=0.05$, $p=0.962$	$t(20)=0.23$, $p=0.823$
	successes: T3	$t(18)=0.65$, $p=0.527$	$t(16)=0.52$, $p=0.607$
	successes: T4	$t(12)=1.36$, $p=0.199$	$t(8)=0.77$, $p=0.465$
	successes: T5	$t(14)=0.30$, $p=0.768$	$t(9)=0.12$, $p=0.909$
	successes: T6	$t(15)=0.07$, $p=0.943$	*$t(13)=2.12$, $p=0.054$*
	successes: T7	$t(22)=0.39$, $p=0.699$	$t(21)=0.75$, $p=0.461$
	drops: T1	$t(15)=1.16$, $p=0.265$	$t(13)=1.29$, $p=0.221$
	drops: T2	$t(20)=0.26$, $p=0.801$	$t(20)=0.84$, $p=0.411$
	drops: T3	$t(18)=0.49$, $p=0.632$	$t(16)=0.37$, $p=0.715$
	drops: T4	$t(12)=0.88$, $p=0.396$	**$t(8)=3.78$, $p=0.005$**
	drops: T5	$t(14)=0.53$, $p=0.605$	$t(9)=0.62$, $p=0.550$

Genotype *t*-test	drops: T6	*t*(15)=1.05, *p*=0.312	*t*(13)=1.34, *p*=0.203
	drops: T7	*t*(22)=0.52, *p*=0.609	*t*(21)=0.29, *p*=0.777
	hits: T1	*t(15)=2.01, p=0.063*	*t*(13)=0.97, *p*=0.352
	hits: T2	*t*(20)=0.74, *p*=0.468	*t*(20)=0.33, *p*=0.743
	hits: T3	***t*(18)=2.77, *p*=0.013**	*t*(16)=0.31, *p*=0.765
	hits: T4	*t*(12)=0.82, *p*=0.430	*t*(8)=0.29, *p*=0.779
	hits: T5	*t*(14)=0.48, *p*=0.638	*t*(9)=0.10, *p*=0.924
	hits: T6	*t*(15)=0.86, *p*=0.406	***t*(13)=2.98, *p*=0.011**
	hits: T7	*t*(22)=0.33, *p*=0.743	*t*(21)=0.55, *p*=0.588
	misses: T1	*t*(15)=1.19, *p*=0.255	***t*(13)=2.16, *p*=0.050**
	misses: T2	*t*(20)=0.04, *p*=0.971	*t*(20)=0.30, *p*=0.769
	misses: T3	*t*(18)=0.51, *p*=0.620	*t*(16)=1.03, *p*=0.317
	misses: T4	*t*(12)=1.37, *p*=0.196	*t*(8)=0.83, *p*=0.430
	misses: T5	*t*(14)=0.57, *p*=0.579	*t*(9)=0.08, *p*=0.937
	misses: T6	*t*(15)=0.29, *p*=0.776	*t*(13)=0.73, *p*=0.480
	misses: T7	*t*(22)=0.63, *p*=0.535	*t(21)=1.76, p=0.093*
	"uncounted" reaches: T1	*t*(15)=0.15, *p*=0.885	*t(13)=2.07, p=0.059*
	"uncounted" reaches: T2	*t*(20)=0.74, *p*=0.465	*t*(20)=0.50, *p*=0.626
	"uncounted" reaches: T3	*t(18)=1.87, p=0.078*	*t*(16)=0.37, *p*=0.720
	"uncounted" reaches: T4	*t*(12)=0.18, *p*=0.862	*t*(8)=0.88, *p*=0.405
	"uncounted" reaches: T5	*t*(14)=0.23, *p*=0.818	*t*(9)=1.52, *p*=0.164
	"uncounted" reaches: T6	*t*(15)=1.19, *p*=0.252	*t*(13)=1.09, *p*=0.298
	"uncounted" reaches: T7	*t*(22)=0.02, *p*=0.984	*t(21)=1.97, p=0.062*
	% FFW: Habituation	*t*(22)=0.34, *p*=0.741	*t*(21)=0.37, *p*=0.713
	% FFW: Shaping 1	*t*(22)=0.33, *p*=0.742	*t*(21)=0.94, *p*=0.360
	% FFW: Shaping 2	*t*(22)=0.17, *p*=0.870	*t(21)=2.04, p=0.054*
	% FFW: T1	*t*(22)=0.50, *p*=0.620	***t*(21)=2.16, *p*=0.042**
	% FFW: T2	*t*(22)=0.16, *p*=0.877	*t*(21)=0.87, *p*=0.394
	% FFW: T3	*t*(22)=0.38, *p*=0.708	*t*(21)=1.56, *p*=0.134
	% FFW: T4	*t*(22)<0.01, *p*>0.999	*t*(21)=0.99, *p*=0.333
	% FFW: T5	*t*(22)=0.42, *p*=0.680	*t*(21)=1.46, *p*=0.159
	% FFW: T6	*t*(22)=0.09, *p*=0.932	***t*(21)=3.51, *p*=0.002**
	% FFW: T7	*t*(22)=0.60, *p*=0.557	***t*(21)=2.15, *p*=0.044**
	FFW	*t*(22)=0.05, *p*=0.959	*t*(21)=0.20, *p*=0.845
	average % FFW	*t*(22)=0.13, *p*=0.899	***t*(21)=2.16, *p*=0.042**

Significant results are in bold, trends are in italics. FFW, free-feeding weight. T, training day.

Table S6. Statistics for Fear Conditioning, related to Figure 5.

Analysis	Effect	Statistics in Females	Statistics in Males
**Weak Trace Fear Conditioning**			
Analysis	Effect	Statistics in Females	Statistics in Males
4 (phase) x 2 (genotype) ANOVA for % freezing	genotype	$F(1,22)=0.07$, $p=0.791$	$F(1,21)=0.52$, $p=0.478$
	phase	**$F(3,66)=43.97$, $p<0.001$**	**$F(3,66)=51.21$, $p<0.001$**
	genotype x phase	$F(3,66)=1.86$, $p=0.145$	$F(3,66)=0.60$, $p=0.620$
Genotype *t*-test	training baseline	$t(22)=0.61$, $p=0.551$	$t(21)=0.42$, $p=0.682$
	context test	$t(22)=1.46$, $p=0.159$	$t(21)=1.05$, $p=0.306$
	cue baseline	$t(22)=0.55$, $p=0.589$	$t(21)=0.07$, $p=0.943$
	cue test	$t(22)=1.10$, $p=0.283$	$t(21)=0.30$, $p=0.771$
baseline vs. test paired-samples *t*-tests: *Pten*$^{+/+}$	training/context	**$t(11)=6.62$, $p<0.001$**	**$t(11)=5.15$, $p<0.001$**
	cue	**$t(11)=8.78$, $p<0.001$**	**$t(11)=8.83$, $p<0.001$**
baseline vs. test paired-samples *t*-tests: *Pten*$^{+/-}$	training/context	**$t(11)=5.17$, $p<0.001$**	**$t(10)=6.12$, $p<0.001$**
	cue	**$t(11)=4.41$, $p=0.001$**	**$t(10)=6.38$, $p<0.001$**
**Remote Memory for Trace Fear Conditioning**			
Analysis	Effect	Statistics in Females	Statistics in Males
4 (phase) x 2 (genotype) ANOVA for % freezing	genotype	$F(1,45)=0.02$, $p=0.902$	**$F(1,67)=4.43$, $p=0.039$**
	phase	**$F(3,135)=51.52$, $p<0.001$**	**$F(3,201)=129.14$, $p<0.001$**
	genotype x phase	$F(3,135)=0.57$, $p=0.637$	**$F(3,201)=2.71$, $p=0.046$**
	post hoc (Sidak): *Pten*$^{+/+}$ vs *Pten*$^{+/-}$	n/a	training baseline $p=0.653$
		n/a	context test **$p=0.032$**
		n/a	cue baseline $p=0.735$
		n/a	cue test $p=0.211$
Genotype *t*-test	training baseline	$t(45)=0.94$, $p=0.354$	$t(67)=0.45$, $p=0.653$
	context test	$t(45)=0.65$, $p=0.523$	**$t(67)=2.19$, $p=0.032$**
	cue baseline	$t(45)=0.83$, $p=0.410$	$t(67)=0.34$, $p=0.735$
	cue test	$t(45)=0.45$, $p=0.656$	$t(67)=1.26$, $p=0.211$
baseline vs. test paired-samples *t*-tests: *Pten*$^{+/+}$	training/context	**$t(24)=6.48$, $p<0.001$**	**$t(33)=14.40$, $p<0.001$**
	cue	**$t(24)=7.45$, $p<0.001$**	**$t(33)=7.28$, $p<0.001$**
baseline vs. test paired-samples *t*-tests: *Pten*$^{+/-}$	training/context	**$t(21)=6.26$, $p<0.001$**	**$t(34)=8.36$, $p<0.001$**
	cue	**$t(21)=4.34$, $p<0.001$**	**$t(34)=7.94$, $p<0.001$**
**Cued Fear Conditioning Extinction**			
Analysis	Effect	Statistics in Females	Statistics in Males
10 (bin) x 2 (genotype) ANOVA for % freezing	genotype	$F(1,44)=0.33$, $p=0.569$	*$F(1,43)=3.44$, $p=0.070$*
	bin	**$F(9,396)=19.08$, $p<0.001$**	**$F(9,387)=13.55$, $p<0.001$**
	genotype x bin	$F(9,396)=0.99$, $p=0.448$	$F(9,387)=0.70$, $p=0.713$
Genotype *t*-test	1st CS	$t(45)=0.39$, $p=0.696$	**$t(46)=2.54$, $p=0.015$**
	last CS	$t(44)=1.45$, $p=0.153$	$t(45)=0.87$, $p=0.391$
	ext score	$t(44)=0.53$, $p=0.598$	$t(43)=0.55$, $p=0.587$
	ext 1 bin 1 (1st bin)	$t(44)=0.05$, $p=0.964$	$t(44)=1.40$, $p=0.168$
	ext 1 bin 2	$t(44)=0.25$, $p=0.801$	$t(44)=1.56$, $p=0.126$
	ext 1 bin 3	$t(44)=0.21$, $p=0.838$	$t(45)=1.27$, $p=0.212$
	ext 1 bin 4	$t(44)=0.19$, $p=0.849$	*$t(45)=1.76$, $p=0.086$*
	ext 1 bin 5	$t(44)=0.18$, $p=0.860$	$t(45)=1.25$, $p=0.216$
	ext 2 bin 1	**$t(45)=2.13$, $p=0.039$**	$t(46)=0.88$, $p=0.384$
	ext 2 bin 2	$t(45)=1.12$, $p=0.267$	$t(45)=1.25$, $p=0.219$
	ext 2 bin 3	$t(45)=0.80$, $p=0.429$	$t(45)=1.66$, $p=0.104$
	ext 2 bin 4	$t(45)=1.18$, $p=0.246$	$t(45)=0.20$, $p=0.845$
	ext 2 bin 5 (last bin)	$t(45)=0.77$, $p=0.443$	$t(45)=0.62$, $p=0.538$
	recall	$t(45)=0.15$, $p=0.881$	$t(45)=0.03$, $p=0.974$
One-way (bin) ANOVA	*Pten*$^{+/+}$	**$F(9,198)=8.36$, $p<0.001$**	**$F(9,189)=8.76$, $p<0.001$**
	Pten$^{+/-}$	**$F(9,198)=12.37$, $p<0.001$**	**$F(9,198)=5.59$, $p<0.001$**

Paired-samples *t*-tests: 1st vs. last bin	*Pten*$^{+/+}$	***t*(22)=2.22, *p*=0.037**	***t*(22)=2.54, *p*=0.018**
	Pten$^{+/-}$	***t*(22)=3.70, *p*=0.001**	*t*(22)=1.16, *p*=0.261
Paired-samples *t*-tests: 1st vs. last CS presentation	*Pten*$^{+/+}$	***t*(22)=4.63, *p*<0.001**	***t*(21)=3.73, *p*=0.001**
	Pten$^{+/-}$	***t*(22)=8.05, *p*<0.001**	***t*(22)=3.06, *p*=0.006**

Significant results are in bold, trends are in italics. CS, conditioned stimulus (cue tone). Ext, extinction.

Table S8. Age of mice (in postnatal days) at testing, related to STAR Methods.

Assay	Number in Battery	Age Range	
		Females	Males
Acoustic Startle Threshold (AST)	1st in Batteries 1, 3, 4; 4th in Battery 2	60-123	56-125
Acoustic Startle Habituation (ASH) + Pre-pulse Inhibition (PPI)	2nd in Batteries 1, 3, 4; 3rd in Battery 2	61-116	57-128
Novel Object Recognition (NOR)	1st in Battery 5; stand-alone test	84-116	52-99
Puzzle Box	1st in Batteries 2, 6; 3rd in Battery 1	64-162	57-99
Open Field Test (OFT)	2nd in Batteries 2, 6; 4th in Battery 1	74-170	71-113
Morris Water Maze (MWM)	2nd in Battery 5; stand-alone test	60-89	52-89
Rotarod Learning	1st in Battery 7	78-128	78-128
Weak Trace Fear Conditioning (WFC)	stand-alone test	120-132	88-131
Remote Memory for Trace Fear Conditioning (RMFC)	2nd in Battery 7; stand-alone test	91-141	73-123
Fear Conditioning Extinction (FCExtn)	stand-alone test	66-118	62-90
Single-Seed Reaching Task (SSRT)	stand-alone test	75-116	68-98

Batteries: 1) AST, ASR+PPI, Puzzle Box, OFT; 2) Puzzle Box, OFT, ASR+PPI, AST; 3) AST, ASR+PPI, SSRT; 4) AST, ASR+PPI; 5) NOR, MWM; 6) Puzzle Box, OFT; 7) Rotarod Learning, RMFC.

Table S9. Schedule and conditions for puzzle box assay, related to Figure 4, STAR Methods.

Day	Trial	Test	Condition	Maximum Duration	Technique Required
1	1	1 (C0T1)	0 (no obstacle)	180 s	n/a (open entry)
	2	1 (C1T1)	1 (tunnel)	240 s	entry through tunnel
	3	2 (C1T2)			
2	1	3 (C1T3)			
	2	1 (C2T1)	2 (tunnel+bedding)	300 s	digging through bedding
	3	2 (C2T2)			
3	1	3 (C2T3)			
	2	1 (C3T1)	3 (tissue)	300 s	pull out tissue OR climb over and push down tissue
	3	2 (C3T2)			
4	1	3 (C3T3)			
	2	1 (C4T1)	4 (foam)	360 s	push foam into goal box
	3	2 (C4T2)			
5	1	3 (C4T3)			
	2	2 (C0T2)	0 (no obstacle)	180 s	n/a (open entry)
